# Structure–Driven Intramolecular Charge Transfer Modulation in Imidazole Fluorophores for Selective Hydrazine Detection and Forensic Applications

**DOI:** 10.1021/acsomega.6c06606

**Published:** 2026-10-01

**Authors:** Yu-Hsin Chen, Yu-Hong Chen, Wei-Ting Chien, Hsin-Chieh Lin, Mei-Yu Yeh

**Affiliations:** † Department of Chemistry, 34900 Chung Yuan Christian University, Taoyuan City, Taiwan 320314, Republic of China; ‡ Department of Materials Science and Engineering, National Yang Ming Chiao Tung University, Hsinchu, Taiwan 300093, Republic of China; § Center for Intelligent Drug Systems and Smart Bio-Devices (IDS^2^B), National Yang Ming Chiao Tung University, Hsinchu, Taiwan 30068, Republic of China

## Abstract

Hydrazine is a highly toxic, potentially carcinogenic compound widely used in industry, necessitating sensitive detection. Meanwhile, latent fingerprint identification requires high-contrast, high-resolution imaging. Both demand rapid and visually discernible signals, motivating the development of integrated materials for simultaneous chemical sensing and forensic imaging. Herein, we report a rationally designed imidazole-based fluorophore, DPI–DCN, that integrates reaction-based hydrazine sensing with high-resolution latent fingerprint (LFP) visualization. By incorporating a strongly electron-withdrawing dicyanovinyl unit, DPI–DCN establishes an efficient donor–acceptor framework, leading to enhanced intramolecular charge transfer (ICT) and pronounced bathochromic shifts relative to its ester analogue, DPI–DET. These photophysical characteristics are supported by density functional theory calculations, which reveal a reduced HOMO–LUMO gap and favorable orbital separation. DPI–DCN exhibits excellent selectivity toward hydrazine, displaying a distinct fluorescence “turn-off” response with a low detection limit of 1.94 μM. Mechanistic investigations using ^1^H NMR and mass spectrometry confirm that nucleophilic addition of hydrazine to the dicyanovinyl moiety disrupts π-conjugation and suppresses ICT, accounting for the observed optical changes. Beyond solution-phase sensing, DPI–DCN functions as an effective solid-state fluorescent powder for LFP development, enabling high-contrast imaging across diverse substrates, including aged fingerprints. Notably, multilevel fingerprint features up to level III are clearly resolved, demonstrating its capability for detailed forensic analysis. This work establishes a direct structure–property–function relationship and presents a versatile molecular platform that bridges chemical sensing and forensic imaging, offering new opportunities for integrated analytical and security applications.

## Graphical abstract

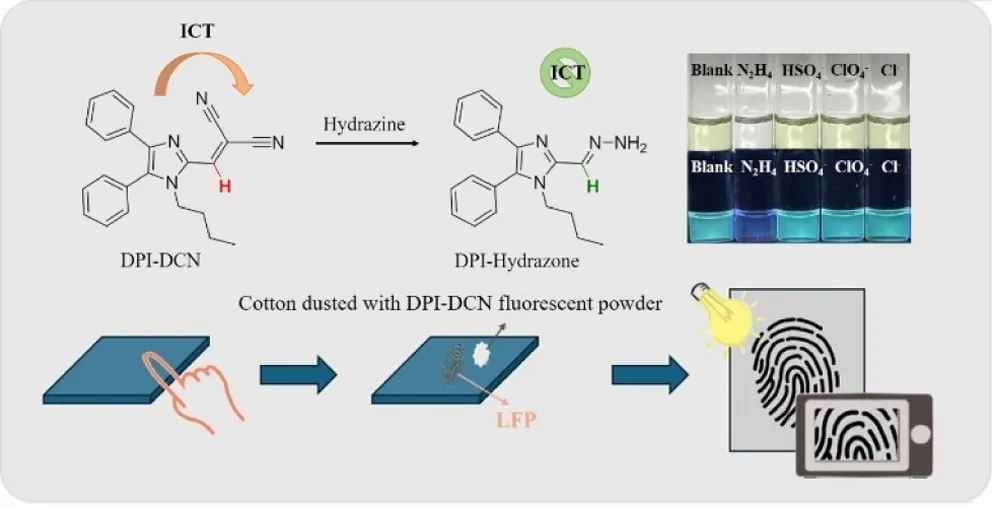

## 1 Introduction

Hydrazine is a colorless and highly toxic compound with an ammonia-like odor, widely used in aerospace propellants, boiler corrosion inhibitors, explosives, and antioxidants.^1–3^ Owing to its strong reducing capability and high reactivity, hydrazine plays a critical role in numerous industrial processes.^4,5^ However, it is also classified as a potential carcinogen and poses serious risks to both environmental and human health. Exposure to hydrazine via inhalation or ingestion can lead to dizziness, nausea, vomiting, chemical burns, and liver dysfunction, while prolonged exposure may result in tumor formation and irreversible damage to the respiratory and central nervous systems. Therefore, the development of highly sensitive and selective detection methods for hydrazine is of considerable importance for industrial safety, environmental monitoring, and public health protection. Conventional methods for hydrazine detection, including chromatographic, electrochemical, and spectroscopic techniques, generally offer good accuracy but often require sophisticated instrumentation, tedious sample preparation, and are not well suited for real-time or on-site analysis.^6–10^ In contrast, fluorescence-based sensing has emerged as a powerful alternative due to its operational simplicity, rapid response, and high sensitivity.^11,12^ In particular, reaction-based fluorescent probes, in which specific recognition sites are incorporated into the molecular framework, enable selective chemical transformations upon interaction with hydrazine, resulting in pronounced optical signal changes and facilitating visual detection.

On the other hand, latent fingerprint (LFP) identification remains indispensable in forensic science for personal identification and crime scene reconstruction.^13–17^ Fingerprints consist of complex mixtures of sebum, sweat, and other biological residues, and are typically invisible without development techniques. Traditional methods, such as powder dusting, iodine fuming, and ninhydrin treatment, are widely used but often suffer from limited contrast, susceptibility to background interference, and restricted substrate compatibility.^18,19^ In recent years, fluorescent materials have gained increasing attention for fingerprint visualization, as they provide high-contrast imaging under UV irradiation and enable the resolution of fine ridge details.^20–23^ Their advantages are particularly evident on complex backgrounds and diverse substrates, where improved clarity and accuracy are required. Notably, although hydrazine sensing and fingerprint visualization belong to distinct application domains, both rely on highly sensitive and selective signal outputs and benefit from rapid and intuitive visual readouts. The integration of these functionalities into a single material platform would therefore be highly advantageous. For example, in forensic scenarios where chemical residues such as hydrazine may be present in fingerprints, a reactive fluorescent material could simultaneously provide chemical information and high-resolution fingerprint images, thereby enhancing analytical efficiency and information integration.

In this context, we report the design and synthesis of two imidazole-based fluorophores, DPI–DCN and DPI–DET, with systematically tuned intramolecular charge transfer (ICT) characteristics (Scheme 1). By incorporating a strongly electron-withdrawing dicyanovinyl unit, DPI–DCN establishes a pronounced donor–acceptor framework, leading to enhanced fluorescence properties upon photoexcitation. Importantly, this moiety also serves as a reactive site for hydrazine, undergoing nucleophilic addition that disrupts π-conjugation and induces significant optical signal changes, thereby enabling highly selective hydrazine detection. In addition, DPI–DCN exhibits favorable solid-state fluorescence and can be directly applied as a powder for latent fingerprint development, affording high-contrast and high-resolution images across various substrates. Through the integration of chemical reactivity and superior photophysical properties, DPI–DCN represents a versatile platform that combines hydrazine sensing with latent fingerprint visualization, offering new opportunities for applications in chemical sensing and forensic science.

**Scheme 1:**
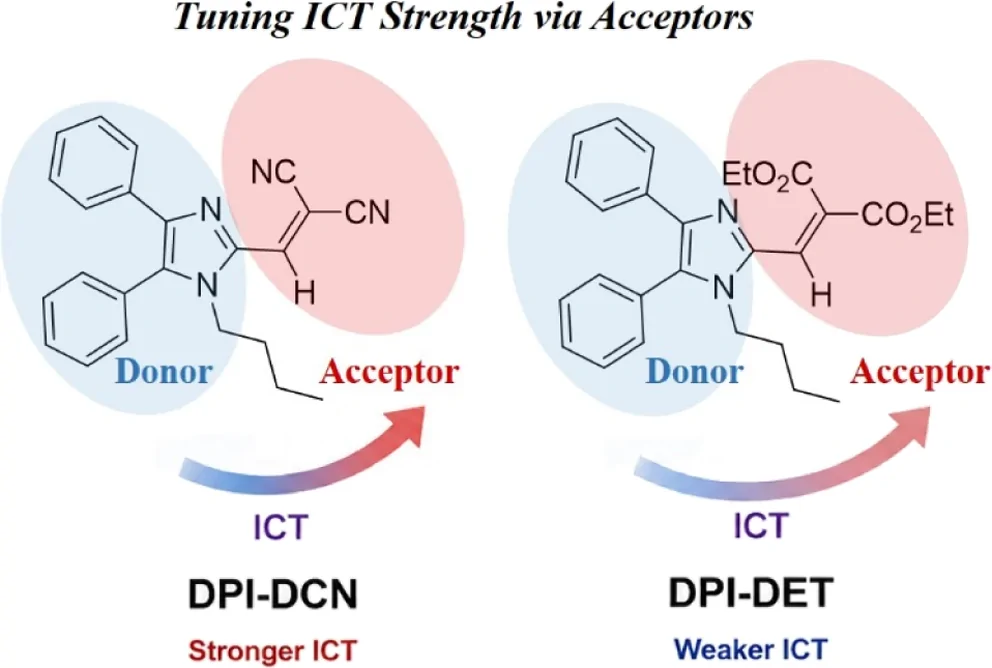
Molecular Design of the Fluorescent Probe; (ICT: Intramolecular Charge Transfer)

## 2 Materials and Methods

### 2.1 Synthesis of 2-((1-Butyl-4,5-diphenyl-1*H*-imidazole-2-yl)­methylene)­malononitrile (DPI–DCN)

The synthesis of DPI–CHO is described in the Supporting Information. DPI–CHO (0.21 g, 0.76 mmol) was dissolved in ethanol (5 mL), followed by the addition of 2–3 drops of piperidine as a catalyst. The reaction mixture was cooled in an ice bath, and malononitrile (0.63 mL, 0.76 mmol) was added dropwise. The reaction was allowed to proceed for 15 min under these conditions. The crude product was purified by reprecipitation from a mixed solvent system of acetone and diethyl ether (1:10, v/v) to afford DPI–DCN as a yellow solid (0.14 g, 54%). ^1^H NMR (400 MHz, DMSO-*d*_6_): δ 8.53 (s, 1H), 7.60–7.62 (m, 3H), 7.42–7.49 (m, 4H), 7.21–7.28 (m, 3H), 4.04 (t, *J* = 7.3 Hz, 2H), 1.41–1.49 (m, 2H), 1.06–1.12 (m, 2H), 0.66 (t, *J* = 7.3 Hz, 3H). ^13^C NMR (100 MHz, DMSO-*d*_6_): δ 142.8, 142.0, 139.3, 136.0, 133.3, 130.8, 130.5, 130.0, 129.4, 128.9, 128.2, 126.8, 115.8, 114.1, 78.1, 44.0, 33.5, 19.4, 13.6. ESI–MS *m*/*z* for C_23_H_21_N_4_: [M + H]^+^ calcd, 353.1766; found, 353.1773.

### 2.2 Synthesis of Diethyl 2-((1-Butyl-4,5-diphenyl-1*H*-imidazole-2-yl)­methylene)-malonate (DPI–DET)

DPI–CHO (1.11 g, 4.02 mmol) was dissolved in toluene (15 mL) in a single-neck round-bottom flask. Piperidine (2–3 drops) was added as a catalyst, followed by the addition of diethyl malonate (0.61 mL, 4.02 mmol). The reaction mixture was heated to reflux at 80 °C and stirred for 16 h. After completion, the crude product was purified by reprecipitation from a mixed solvent system of acetone and diethyl ether (1:10, *v*/*v*) to afford DPI–DET as a pale yellow solid (0.52 g, 30%). ^1^H NMR (400 MHz, DMSO-*d*_6_): δ 7.56–7.58 (m, 4H), 7.42–7.44 (m, 2H), 7.32–7.34 (m, 2H), 7.14–7.24 (m, 3H), 4.24–4.37 (m, 4H), 3.98 (t, *J* = 7.2 Hz, 2H), 1.43–1.50 (m, 2H), 1.26–1.27 (m, 6H), 1.09–1.15 (m, 2H), 0.71 (t, *J* = 7.2 Hz, 3H). ^13^C NMR (100 MHz, DMSO-*d*_6_): δ 165.9, 164.0, 139.7, 139.5, 134.2, 132.7, 131.1, 130.3, 129.8, 129.4, 128.7, 127.3, 126.5, 125.8, 125.4, 111.8, 61.4, 43.4, 33.4, 20.1, 13.5, 13.1. ESI–MS *m*/*z* for C_27_H_30_N_2_NaO_4_: [M + Na]^+^ calcd, 469.2103; found, 469.2102.

### 2.3 Characterizations

^1^H and ^13^C NMR spectra were acquired on a Bruker Avance NEO 400 MHz spectrometer with DMSO-*d*_6_ as the solvent. UV–vis absorption measurements were performed using a Shimadzu UV-2550 spectrophotometer, while fluorescence emission spectra were recorded on a Horiba FluoroMax-4 spectrofluorometer. The emission profiles of DPI–DCN and DPI–DET were collected under excitation wavelengths of 420 and 368 nm, respectively. To minimize inner-filter effects and reabsorption of emitted light, measurements were carried out using micro fluorescence cuvettes with a 1 mm optical path length and a total volume of 0.35 mL.

### 2.4 Computational methods

All quantum chemical calculations were carried out using the Gaussian 98 program package.^24^ The molecular geometries of DPI–DCN and DPI–DET were fully optimized employing density functional theory (DFT) with the long-range-corrected CAM-B3LYP functional and the 6–31 + G­(d,p) basis set.^25^ To account for solvent effects, the polarizable continuum model (PCM) was employed, and calculations were performed for *n*-hexane, THF, and DMSO. The inclusion of diffuse functions in the 6–31 + G­(d,p) basis set and the long-range correction of CAM-B3LYP were adopted to provide a more appropriate description of the donor–acceptor electronic structures and ICT characteristics of the investigated molecules.^25–27^ The optimized geometries were verified by vibrational frequency calculations, and no imaginary frequencies were observed, confirming that the optimized structures correspond to true minima on the respective potential energy surfaces. Frontier molecular orbital analysis, including the highest occupied molecular orbital (HOMO) and lowest unoccupied molecular orbital (LUMO), was conducted to elucidate the ICT characteristics and to identify the regions involved in electronic transitions within the molecules. Time-dependent DFT (TD-DFT) calculations were further employed to estimate the electronic excitation energies and corresponding absorption and emission wavelengths.

## 3 Results and Discussion

### 3.1 Material Design and Photophysical Properties

The synthetic routes of DPI–DCN and DPI–DET are illustrated in Scheme S1. 4,5-Diphenylimidazole was first subjected to *N*-alkylation to introduce a butyl substituent, thereby enhancing solubility. Subsequent formylation afforded the corresponding aldehyde intermediate, which serves as a key handle for further functionalization. Finally, Knoevenagel condensation reactions with malononitrile and diethyl malonate, respectively, were performed under piperidine-catalyzed conditions to successfully afford the target products DPI–DCN and DPI–DET (see Supporting Information for details). The absorption and fluorescence behaviors of the two molecules in dimethyl sulfoxide (DMSO) were further investigated. As shown in Figure 1, DPI–DCN exhibits an absorption maximum (λ_max_) at 420 nm and a corresponding emission maximum at 493 nm upon excitation, giving rise to blue–green fluorescence. In contrast, DPI–DET displays absorption and emission maxima at 368 and 453 nm, respectively, corresponding to blue emission. Notably, DPI–DCN shows a pronounced bathochromic shift in both absorption and emission relative to DPI–DET. This spectral red shift can be attributed to differences in ICT character.^28–30^ The terminal dicyano group in DPI–DCN is a significantly stronger electron-withdrawing unit than the diester moiety in DPI–DET, thereby promoting more efficient donor–acceptor charge separation in the excited state. As a result, DPI–DCN exhibits an enhanced ICT character and a reduced HOMO–LUMO energy gap, leading to red-shifted optical transitions. From a structural perspective, the dicyano group in DPI–DCN not only exhibit strong electron-withdrawing ability but also help extend the overall π-conjugation system, increasing the degree of electron delocalization and further lowering the excited-state energy. In contrast, the diester group in DPI–DET, while electron-withdrawing, is less effective in both inductive and resonance interactions, resulting in a weaker ICT effect. To experimentally assess the ICT character, the absorption and fluorescence spectra of both compounds were further measured in solvents with different polarities, including *n*-hexane, THF, and DMSO (Figure S1). DPI–DCN exhibits progressive bathochromic shifts in both absorption and emission with increasing solvent polarity, with absorption maxima at 405, 416, and 420 nm and emission maxima at 435, 451, and 493 nm in *n*-hexane, THF, and DMSO, respectively. Notably, the fluorescence maximum undergoes a 58 nm red shift from *n*-hexane to DMSO, accompanied by a 15 nm shift in the absorption maximum. DPI–DET displays a similar but less pronounced solvent-dependent response, with absorption maxima at 358, 361, and 365 nm and emission maxima at 409, 437, and 453 nm in *n*-hexane, THF, and DMSO, respectively. The corresponding red shifts of 7 nm in absorption and 44 nm in emission are substantially smaller than those observed for DPI–DCN. The progressively red-shifted emission with increasing solvent polarity is consistent with preferential stabilization of the more polar excited state, providing experimental support for an ICT-mediated excited-state process.^31–33^ These results provide experimental evidence that the distinct acceptor groups effectively modulate the ICT character of the two fluorophores, with the dicyanovinyl group in DPI–DCN promoting substantially stronger ICT than the diester group in DPI–DET. To further quantify the emissive properties, the fluorescence quantum yields (Φ_F_) of DPI–DCN and DPI–DET were determined in both solution and solid states (Table S1). In DMSO, DPI–DCN and DPI–DET exhibit Φ_F_ values of 1.02% and 1.86%, respectively. Although DPI–DCN displays a slightly lower fluorescence quantum yield in solution, its emission efficiency is markedly enhanced in the solid state, reaching 13.53%, compared with 6.27% for DPI–DET. The substantial enhancement in Φ_F_ upon going from solution to the solid state suggests that aggregation or molecular packing effectively suppresses nonradiative relaxation pathways, particularly for DPI–DCN. This behavior is consistent with the stronger ICT character and rigidified π-conjugated framework of DPI–DCN, which favor radiative emission upon aggregation. Importantly, the approximately 2-fold higher solid-state quantum yield of DPI–DCN relative to DPI–DET provides a quantitative basis for its stronger fluorescence intensity and higher contrast in latent fingerprint visualization.

**Figure 1:**
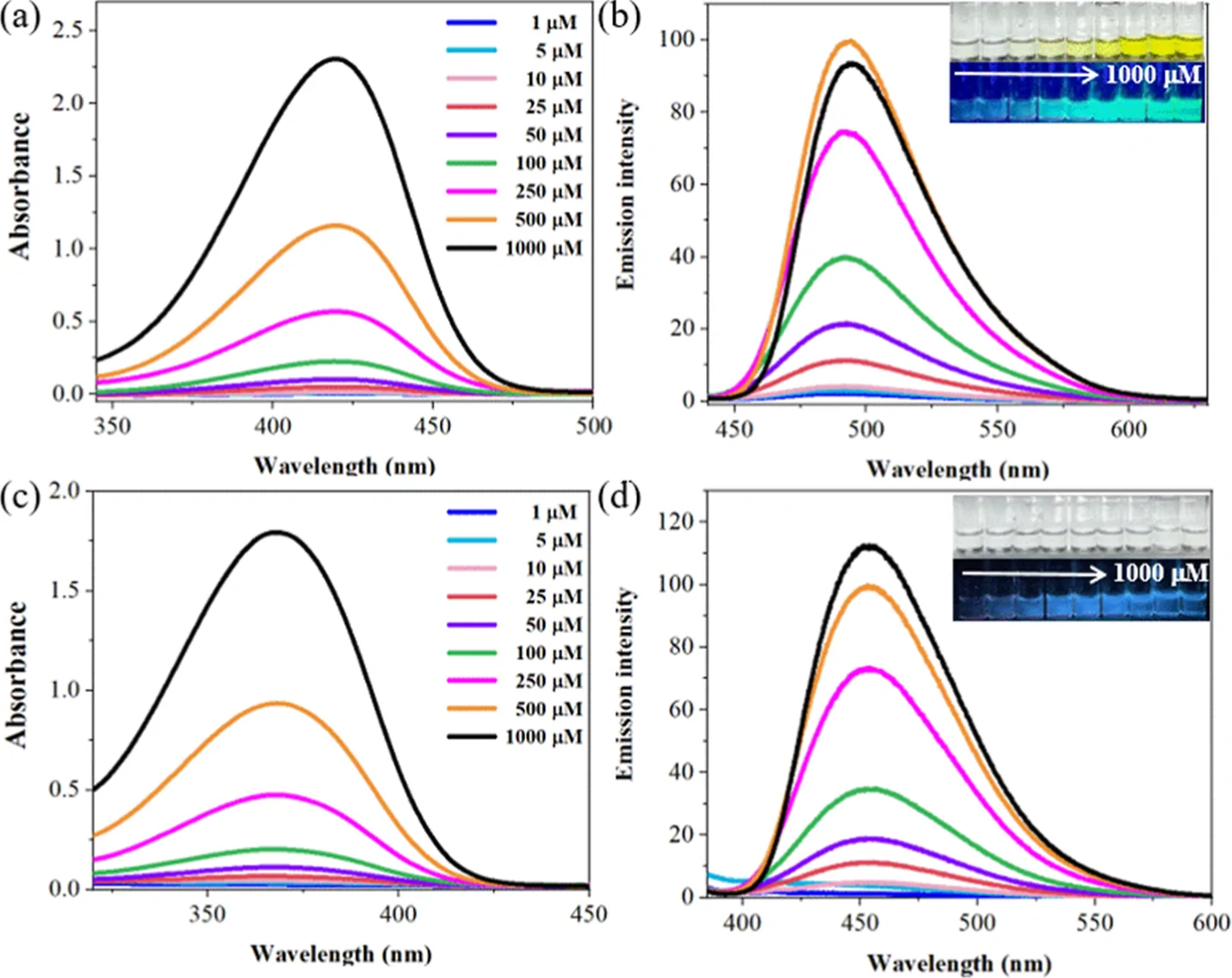
(a) Absorption spectra of DPI–DCN at various concentrations in DMSO, and (b) the corresponding fluorescence spectra. (c) Absorption spectra of DPI–DET at various concentrations in DMSO, and (d) the corresponding fluorescence spectra. (DPI–DCN: λ_ex_ = 420 nm; DPI–DET: λ_ex_ = 368 nm. Insets show the optical images of the solutions at each concentration, with the upper panels taken under ambient light and the lower panels under 365 nm UV irradiation.).

### 3.2 Theoretical Calculations

To further validate the proposed correlation between ICT character and the observed bathochromic shift, quantum chemical calculations were performed for DPI–DCN and DPI–DET using the long-range-corrected CAM-B3LYP/6–31 + G­(d,p) level in conjunction with the polarizable continuum model (PCM) to account for solvent effects.^25–27^ Calculations were carried out in *n*-hexane, THF, and DMSO to directly compare the theoretical results with the experimental solvent-dependent photophysical properties. As shown in Figure S2, the calculated HOMO–LUMO energy gaps of both compounds exhibit a systematic dependence on solvent polarity, with the energy gap decreasing as the solvent polarity increases. Notably, DPI–DCN consistently possesses a smaller HOMO–LUMO gap than DPI–DET in all three solvents, consistent with its stronger donor–acceptor character and experimentally observed red-shifted optical transitions. To further establish the relationship between the calculated electronic structures and the experimental optical properties, the absorption and emission wavelengths were calculated using TD-DFT and compared with the corresponding experimental values (Table S2). Although the calculated absolute wavelengths do not exactly reproduce the experimental values, the theoretical results successfully reproduce the same solvent-dependent trends observed experimentally for both DPI–DCN and DPI–DET. To provide a molecular-level interpretation of the experimentally observed photophysical properties in DMSO, the electronic structures of DPI–DCN and DPI–DET were further analyzed under the DMSO solvation environment, as presented in Figure 2. Analysis of the orbital distributions reveals that the HOMO of DPI–DCN is primarily delocalized over the conjugated backbone, whereas the LUMO is predominantly localized on the dicyano terminus, consistent with an efficient ICT process. In contrast, DPI–DET exhibits less pronounced LUMO localization on the diester moiety, indicative of less efficient charge separation and a weaker ICT character, resulting in a larger energy gap (Figure 2a). To further visualize the electronic distribution and molecular polarization, electrostatic potential maps (EPM) were also computed (Figure 2b).^34,35^ DPI–DCN displays a markedly electron-deficient region localized at the dicyanovinyl unit, particularly around the vinylic proton, reflecting significant electron withdrawal by the −CN groups and a pronounced polarization of the molecular framework. By comparison, the diester group in DPI–DET, while electron-withdrawing, induces a less significant perturbation, as evidenced by the more modest potential variation in its EPM. Taken together, the HOMO–LUMO energy levels, orbital distributions, and EPM analyses consistently demonstrate that the enhanced ICT character and reduced energy gap of DPI–DCN originate from the strongly electron-withdrawing dicyano unit. This functionality effectively lowers the LUMO energy and facilitates charge separation, thereby accounting for the observed bathochromic shifts in both absorption and emission. The strong agreement between theoretical and experimental results underscores the validity of the molecular design strategy and highlights the utility of theoretical calculations in elucidating structure–property relationships at the molecular level, providing valuable guidance for the rational design of functional optoelectronic and sensing materials.

**Figure 2:**
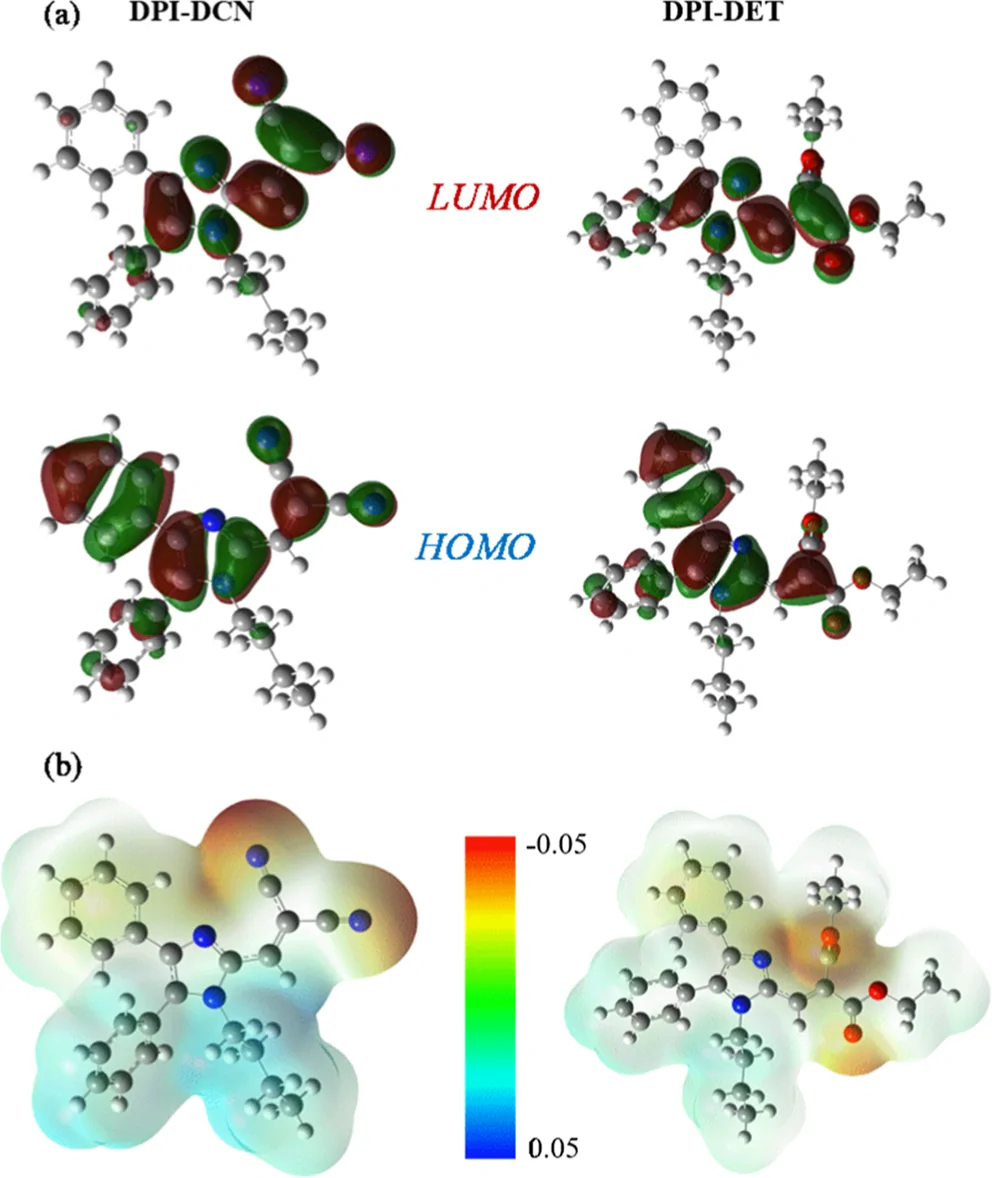
(a) Molecular orbital amplitude plots of HOMO and LUMO of DPI–DCN and DPI–DET. (b) 3D representation of the electrostatic potentials around the molecules of DPI–DCN and DPI–DET. The red surface indicates a region of negative electrostatic potential, while the blue surface represents a region of positive electrostatic potential, with a range from −0.05 to 0.05. Carbon, hydrogen, nitrogen, and oxygen atoms are depicted in gray, white, blue, and red, respectively.

### 3.3 Hydrazine Detection

The selective recognition behavior of DPI–DCN and DPI–DET toward hydrazine and a range of potential interfering species was systematically investigated by monitoring changes in solution color, absorption, and fluorescence spectra. In the initial screening, hydrogen sulfate (HSO_4_^–^), perchlorate (ClO_4_^–^), and chloride (Cl^–^) were selected as representative common inorganic anions to evaluate the susceptibility of the probes to anionic interference. As presented in Figure 3a,b, DPI–DCN exhibits a characteristic absorption band at 420 nm and an emission maximum at 493 nm in the absence of analytes (blank). The solution appears pale yellow under ambient light and displays bright blue–green fluorescence under 365 nm UV irradiation. Upon addition of HSO_4_^–^, ClO_4_^–^, or Cl^–^, both the absorption and emission spectra remain essentially unchanged, indicating negligible interaction with these common anions. In sharp contrast, the introduction of hydrazine induces pronounced spectral changes. The absorption band at 420 nm nearly disappears, suggesting a substantial perturbation of the molecular electronic structure. Concurrently, the fluorescence emission at 493 nm is significantly diminished and is nearly completely quenched. Visually, the solution changes from pale yellow to nearly colorless, and the blue–green fluorescence under 365 nm UV irradiation is effectively extinguished, yielding a dark or nonemissive state. To more rigorously assess the selectivity of DPI–DCN, particularly against competing nucleophiles, the sensing response was further evaluated using hydroxylamine (NH_2_OH), ammonia (NH_3_), methylamine, ethylenediamine, phenylhydrazine, and 2,4-dinitrophenylhydrazine (Figure 3c,d).^36,37^ These analytes were selected to represent structurally and chemically diverse nucleophilic species, including hydroxylamine, ammonia, aliphatic amines, and substituted hydrazines. Notably, none of these potential interferents produced appreciable changes in the absorption or fluorescence spectra of DPI–DCN under identical conditions, whereas hydrazine induced a distinct and pronounced response. This result indicates that the optical response of DPI–DCN is not simply associated with nonspecific nucleophilic attack but is strongly dependent on the molecular structure and reactivity of hydrazine. In contrast, DPI–DET does not exhibit comparable selectivity under identical conditions (Figure S3). Minimal changes in both absorption and fluorescence spectra are observed regardless of the analyte introduced, indicating a lack of effective recognition toward hydrazine. This disparity likely arises from differences in the electron-accepting substituents and overall molecular electronic structures, which influence the reactivity and interaction mechanisms with hydrazine.

**Figure 3:**
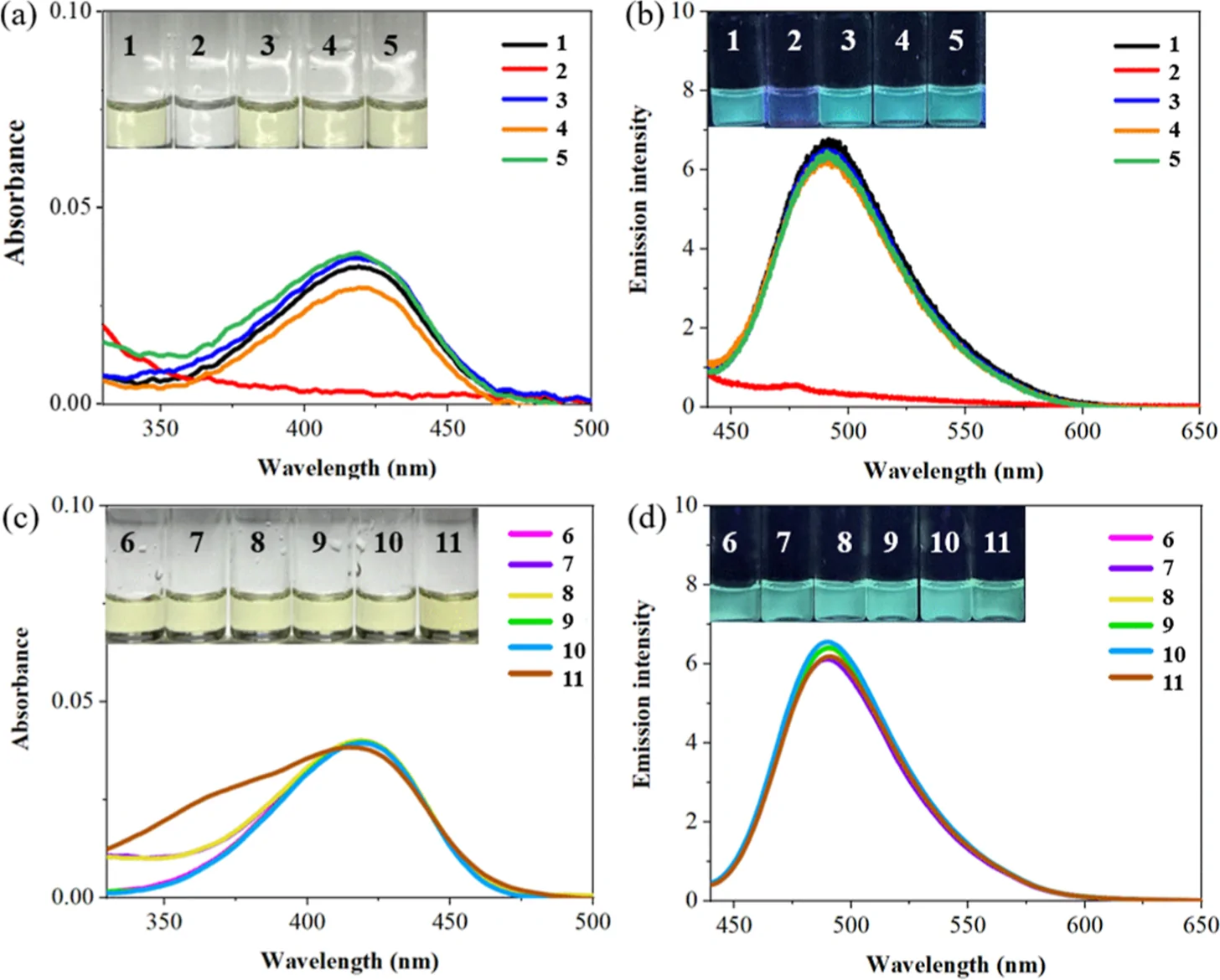
(a,c) Absorption spectra and (b,d) fluorescence spectra of DPI–DCN in the presence of 1 equiv of various analytes: 1, blank; 2, N_2_H_4_; 3, HSO_4_^–^; 4, ClO_4_^–^; 5, Cl^–^; 6, NH_2_OH; 7, NH_3_; 8, methylamine; 9, ethylenediamine; 10, phenylhydrazine; and 11, 2,4-dinitrophenylhydrazine. (λ_ex_ = 420 nm; insets show the corresponding optical images of the solutions before and after the addition of each analyte, with the left panels taken under ambient light and the right panels under 365 nm UV irradiation.).

To further evaluate the sensitivity of DPI–DCN toward hydrazine, concentration-dependent studies were performed in DMSO using a fixed probe concentration of 50 μM. Incremental amounts of hydrazine were added to establish the concentration–response relationship and to determine the limit of detection (LOD).^38–40^ The equivalents of hydrazine were varied systematically from 1 to 0 equiv (blank), including 1, 0.8, 0.5, 0.1, 0.05, 0.01, 0.005, 0.001, 0.0009, 0.0008, 0.0007, 0.0006, 0.0005, 0.0004, 0.0003, 0.0002, and 0.0001 equiv, allowing detailed examination of the low-concentration regime. As shown in Figure 4a,b, a gradual and continuous decrease in the characteristic absorption band at 420 nm is observed with increasing hydrazine concentration. Concurrently, the fluorescence emission at 493 nm diminishes progressively, ultimately leading to pronounced quenching. This concentration-dependent spectral response indicates a well-defined quantitative relationship between DPI–DCN and hydrazine, supporting its applicability for analytical detection. Consistent with the spectroscopic results, visible color changes under ambient light and fluorescence variations under 365 nm UV irradiation are also observed as a function of hydrazine concentration, enabling both instrument-based quantification and rapid visual detection. Based on the linear response region, the LOD was calculated to be 1.94 μM, demonstrating the high sensitivity of DPI–DCN toward hydrazine (Figure 4c). This result highlights the capability of the probe to produce discernible signals even at low analyte concentrations, underscoring its potential for detecting trace levels of hydrazine in environmental and industrial samples.

**Figure 4:**
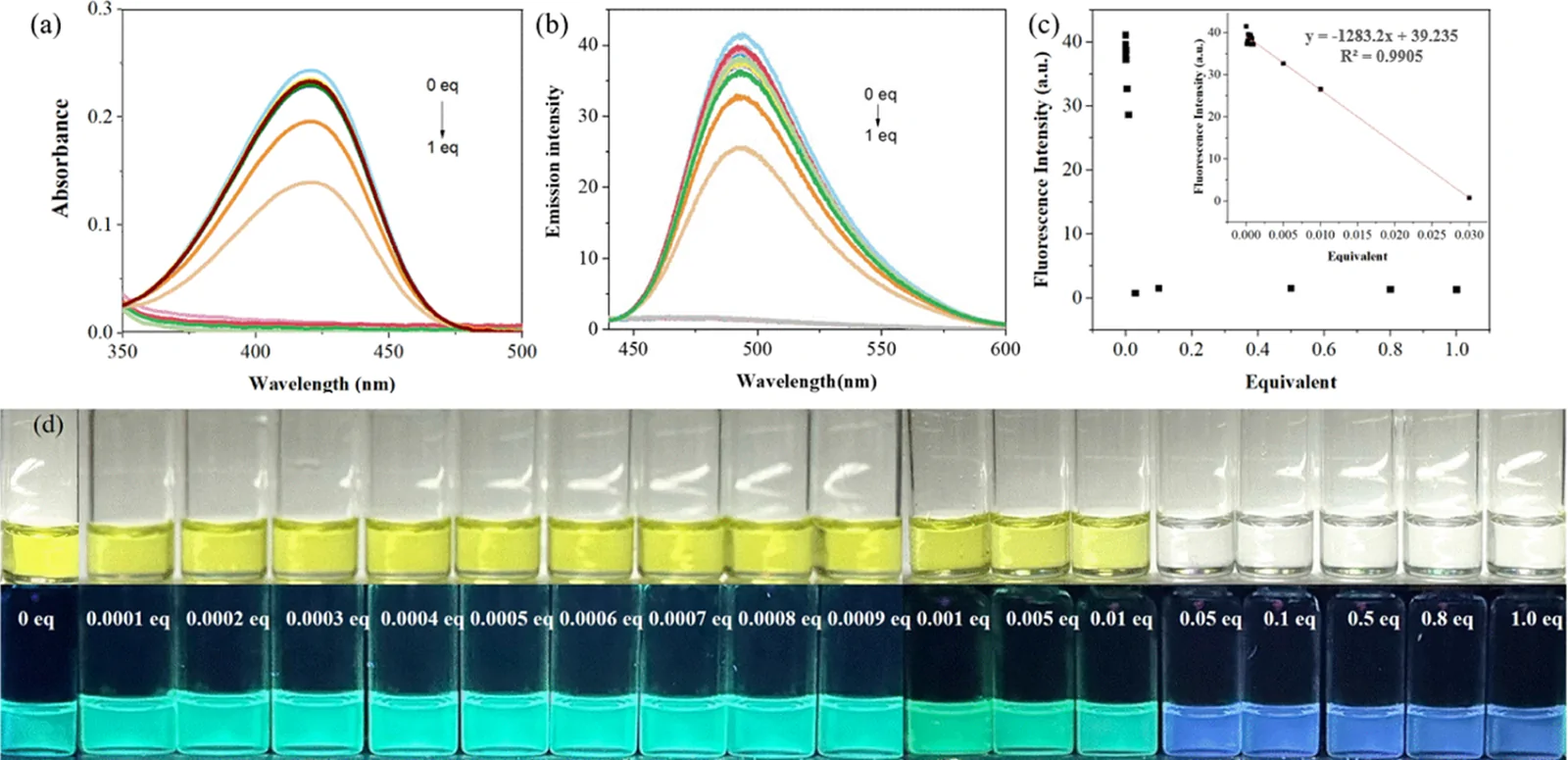
(a) Absorption spectra and (b) fluorescence spectra of DPI–DCN (50 μM in DMSO; λ_ex_ = 420 nm) in the presence of varying equivalents of hydrazine (0–1 equiv); (c) fluorescence intensity versus hydrazine concentration showing the linear relationship and the corresponding LOD; (d) optical images of the solutions under the same conditions, with the upper row recorded under ambient light and the lower row under 365 nm UV irradiation.

To further elucidate the sensing mechanism, we propose that the interaction between DPI–DCN and hydrazine proceeds predominantly via a nucleophilic addition pathway.^41,42^ Owing to its nucleophilicity, hydrazine is expected to attack the electron-deficient dicyanovinyl moiety, thereby disrupting the π-conjugated framework and consequently suppressing the ICT process. This structural perturbation directly affects the electronic transitions, leading to a pronounced decrease in the characteristic absorption band and substantial fluorescence quenching, as shown in Figure 4a,b. Such a reaction-based “turn-off” mechanism accounts for the high selectivity and distinct optical response of DPI–DCN toward hydrazine (Figure 5a). To substantiate this hypothesis, structural characterization was carried out using ^1^H NMR spectroscopy and mass spectrometry. As revealed in Figure 5b, prior to hydrazine addition, the vinylic proton (=CH) of the dicyanovinyl group (highlighted in red) appears at δ = 8.5 ppm, indicative of a highly deshielded electronic environment. Upon treatment with hydrazine, this signal shifts markedly to δ = 7.8 ppm, reflecting a significant change in its chemical environment. This shift is consistent with the corresponding vinylic proton (=CH, highlighted in green) in the proposed hydrazone product (DPI-hydrazone), providing indirect evidence that the original dicyanovinyl moiety participates in the reaction and is transformed into a new functional group. Moreover, the magnitude of the chemical shift change suggests a redistribution of electron density, supporting the attenuation of the ICT effect. Further confirmation was obtained from electrospray ionization mass spectrometry (ESI–MS) analysis (Figure 5c), which reveals a molecular ion peak consistent with the expected mass of the DPI-hydrazone derivative. The agreement between the NMR and MS results provides strong support for the proposed reaction mechanism.

**Figure 5:**
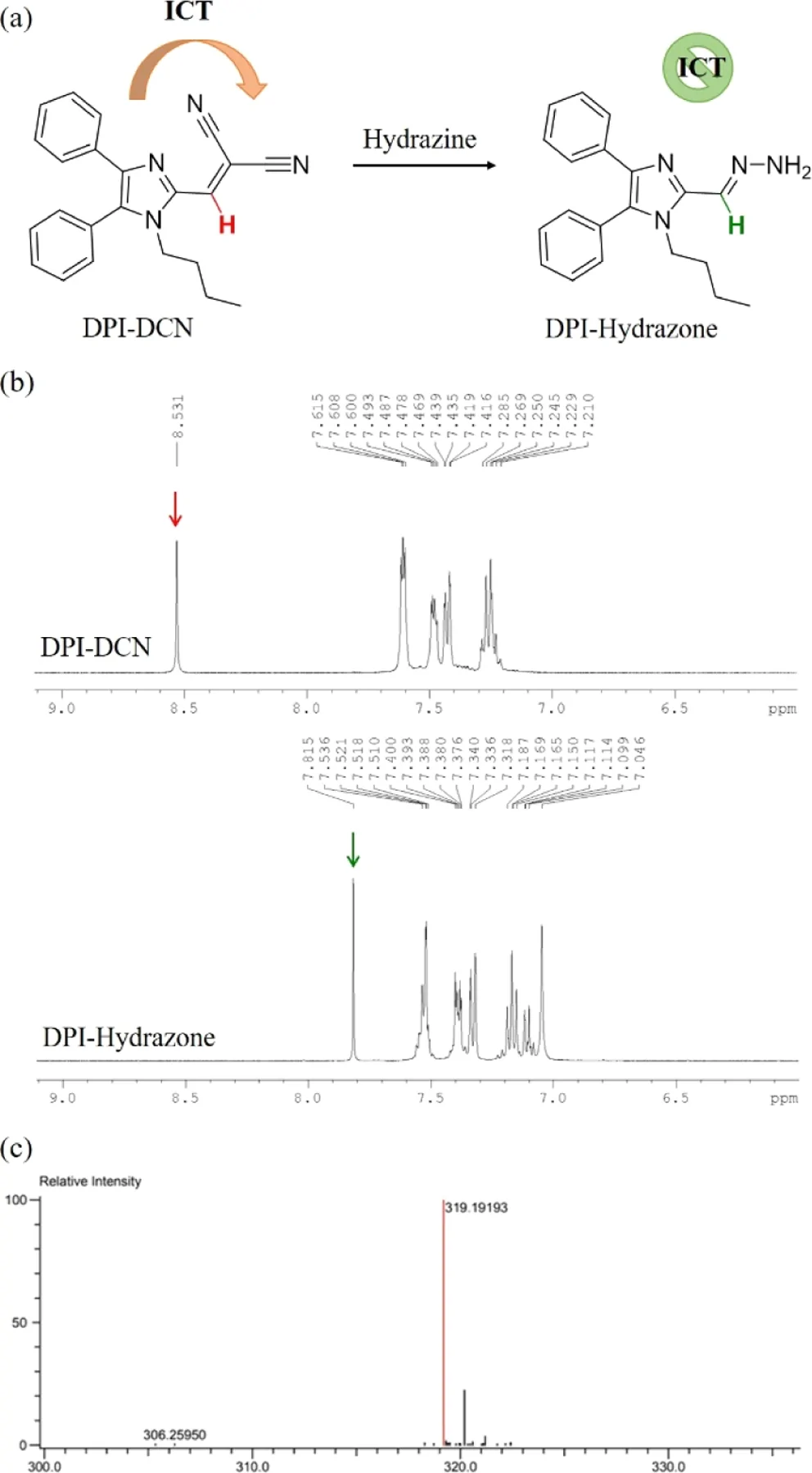
(a) Proposed sensing mechanism of DPI–DCN toward hydrazine; (b) ^1^H NMR spectra of DPI–DCN before (top) and after (bottom) the addition of hydrazine; (c) ESI–MS spectrum of DPI–DCN after reaction with hydrazine.

### 3.4 Forensic Application

The solid-state optical properties of DPI–DCN and DPI–DET, as well as their potential applications in latent fingerprint (LFP) visualization, were further investigated.^43–46^ As shown in Figure 6a,b, the two compounds exhibit distinct differences in both appearance and fluorescence behavior. DPI–DCN appears as a bright yellow powder under ambient light and displays intense and uniform yellow fluorescence under UV irradiation. In contrast, DPI–DET presents a pale yellow color under ambient light and emits blue fluorescence under UV light, albeit with lower overall brightness and contrast. Given their emissive properties, both compounds were evaluated as fluorescent powders for latent fingerprint development. The performance of fingerprint visualization is strongly influenced by the nature of the substrate, particularly its porosity, as well as background color and texture, which can affect image clarity and contrast. To comprehensively assess their applicability, eight commonly encountered substrates were selected, including four nonporous surfaces—glass slides, aluminum foil, polyethylene (PE) plastic bags, and iron plates (Figure 6c)—and four porous materials—banknotes, paper cups, polypropylene (PP) caps, and standard paper (Figure 6d). Experimental results demonstrate that DPI–DCN exhibits excellent fingerprint development performance across all tested substrates. The powder adheres effectively to fingerprint residues, including sebaceous and sweat components, on both smooth nonporous and absorbent porous surfaces, producing high-contrast and well-defined ridge patterns. Notably, even on substrates with complex backgrounds or significant visual interference, DPI–DCN maintains clear and discernible fingerprint features, indicating strong resistance to background interference. In contrast, DPI–DET shows significantly inferior performance, particularly on porous substrates, where fingerprint patterns are barely observable. This diminished performance is likely attributable to its lower fluorescence intensity and/or weaker interactions with fingerprint residues.

**Figure 6:**
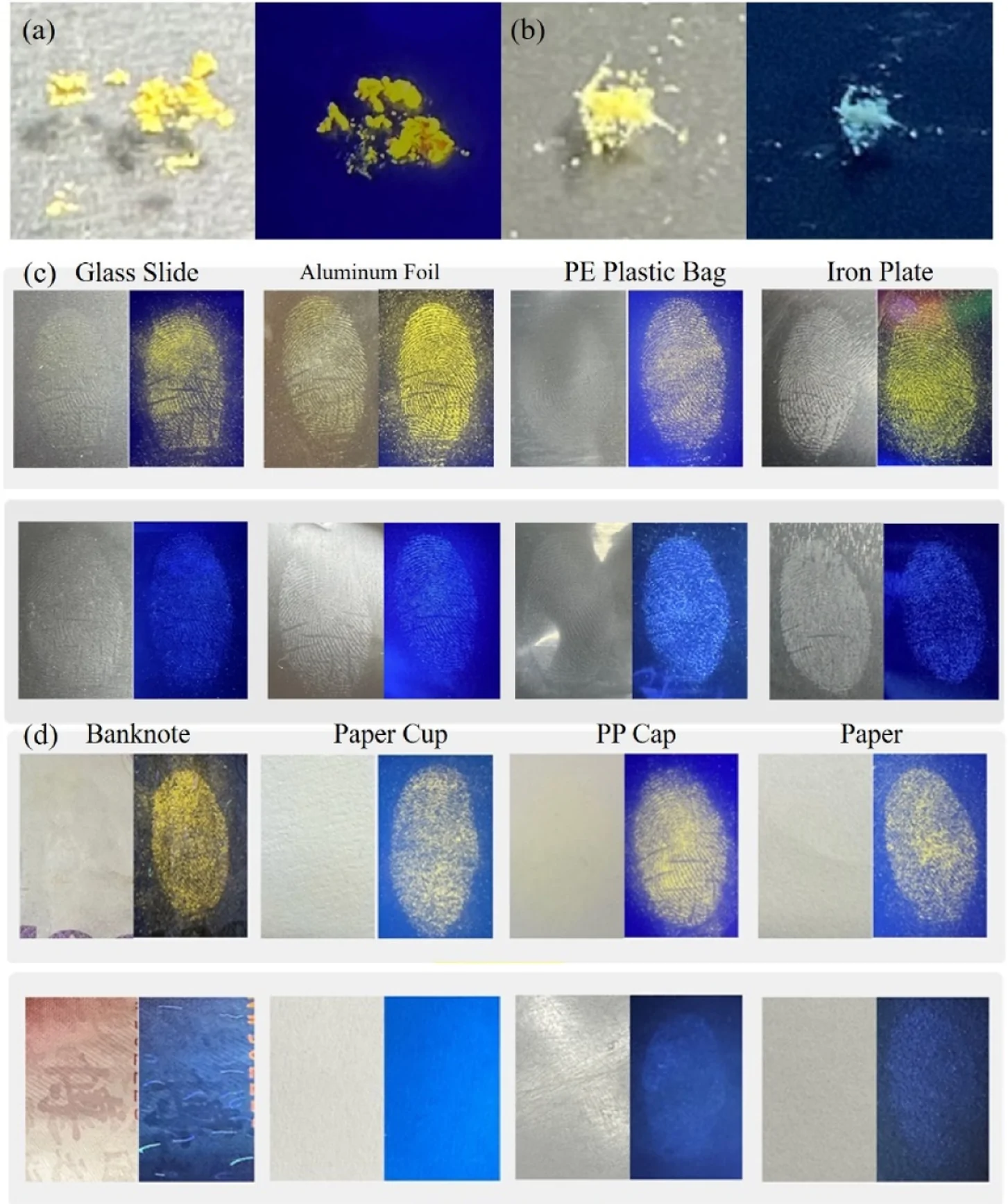
(a) Solid-state image of DPI–DCN; (b) solid-state image of DPI–DET; (c) latent fingerprint images developed using DPI–DCN (top) and DPI–DET (bottom) on nonporous substrates; (d) latent fingerprint images developed using DPI–DCN (top) and DPI–DET (bottom) on porous substrates. In each case, the left panels show observations under ambient light, and the right panels show fluorescence images under 365 nm UV irradiation.

On the basis of the above results, DPI–DCN was selected as the primary material for subsequent fingerprint analysis due to its superior development performance and stability. In forensic investigations, successful visualization must be complemented by reliable feature extraction and comparison. Fingerprint characteristics are conventionally categorized into three hierarchical levels according to their structural information. Level I features describe the overall ridge pattern, such as arches, loops, and whorls, and provide the fundamental classification of a fingerprint. Level II features correspond to minutiae, including ridge endings, bifurcations, dots, islands, short ridges, enclosures, crossovers, and related ridge events, which are widely used for individual identification. Level III features represent finer ridge-level details, such as sweat pores, ridge-edge contours, and other microscopic ridge characteristics, and can provide additional discriminative information when image quality and resolution are sufficient. As displayed in Figure 7, fingerprints developed on aluminum foil using DPI–DCN enable clear identification of multilevel features.^47–52^ At the level I scale, the overall fingerprint pattern is readily discernible, including the core and delta. At the Level II scale, characteristic minutiae such as crossovers, returns, dots, breaks, terminations, and bifurcations can be clearly resolved. Notably, level III features, including creases, sweat pores, and ridge contours, are also distinctly observable. Importantly, DPI–DCN allows visualization of even finer level III details, such as variations in bifurcation angles, geometric parameters of deltas, and crease widths, underscoring its capability for high-resolution fingerprint analysis (Figure 7). In addition, grayscale intensity profiling along selected ridge paths reveals well-defined ridge–valley contrast, enabling quantitative assessment of topographical variations and further enhancing analytical reliability (Figure S4).^53,54^ To further evaluate practical applicability, the stability and persistence of fingerprint visualization were also examined over extended periods.^54,55^ Remarkably, latent fingerprints aged for up to four months could still be successfully developed using DPI–DCN (Figure S5), demonstrating its robustness and effectiveness for the detection of aged fingerprints. To benchmark the performance of DPI–DCN against previously reported fluorescent fingerprint-development materials, a comparative summary is provided in Table S3. Representative fluorescent systems, including organic fluorophores, fluorescent carbon dots, and magnetic or composite materials, have been reported to enable visualization of level I–III fingerprint features; however, their long-term development performance is either limited or not reported in several cases.^56–66^ In comparison, DPI–DCN combines a simple powder-dusting protocol with level I–III fingerprint visualization and maintains effective development of latent fingerprints for at least four months. These results demonstrate that DPI–DCN not only affords robust and high-contrast fingerprint development across diverse substrates but also provides high-resolution structural information essential for forensic identification. This combination of sensitivity, resolution, and stability highlights its strong potential for applications in forensic science and security screening.

**Figure 7:**
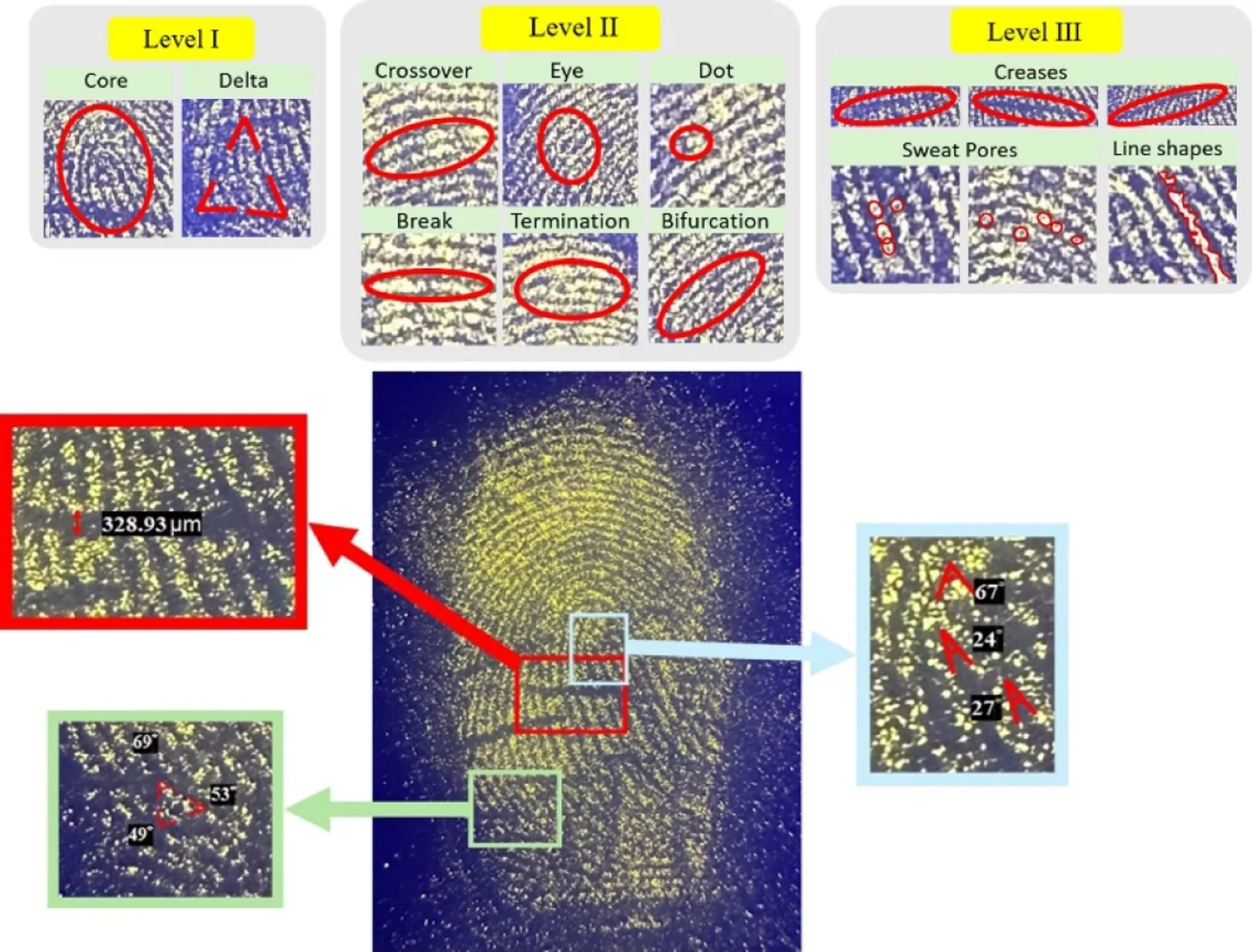
Level I–III feature analysis of latent fingerprints developed using DPI–DCN.

## 4 Conclusions

We report a dicyano-functionalized imidazole fluorophore, DPI–DCN, that integrates sensitive chemical sensing with high-resolution forensic imaging. Compared with the diester analogue DPI–DET, the incorporation of a strong electron-withdrawing dicyano unit enhances ICT, resulting in a reduced HOMO–LUMO gap and pronounced bathochromic shifts in both absorption and emission. These trends are well supported by DFT calculations, including Frontier orbital analysis and electrostatic potential mapping. DPI–DCN exhibits excellent selectivity toward hydrazine, showing a distinct reaction-based fluorescence “turn-off” response with a low detection limit of 1.94 μM. Mechanistic studies using ^1^H NMR and mass spectrometry confirm that nucleophilic addition of hydrazine to the dicyanovinyl group disrupts π-conjugation and suppresses ICT, accounting for the observed optical changes. Importantly, DPI–DCN also performs effectively as a solid-state fluorescent powder for latent fingerprint visualization, delivering high-contrast and durable imaging across diverse substrates, including aged samples. Clear resolution of hierarchical fingerprint features up to Level III further highlights its capability for detailed forensic analysis. This study establishes a clear structure–property–function relationship and demonstrates the potential of DPI–DCN as a versatile platform for both chemical sensing and forensic applications.

## Supplementary Material



## Notes

Scheme S1 synthetic routes; Figure S1 solvent-dependent photophysical properties of DPI–DCN and DPI–DET; Figure S2 calculated HOMO–LUMO energy gaps; Figure S3 absorption spectra and fluorescence spectra of DPI–DET in the presence of 1 equiv of various analytes; Figure S4 grayscale values; Figure S5 fluorescence images of LFPs; Figures S6 and S7 NMR spectra; Figures S8 and S9 HRMS; Table S1 the quantum yields Table S2 experimental and calculated photophysical properties of DPI–DCN and DPI–DET in *n*-hexane, THF, and DMSO; Table S3 comparative performance of DPI–DCN and previously reported fluorescent materials for latent fingerprint development (PDF)
