# Supplementary material for: Structure–Driven Intramolecular Charge Transfer Modulation in Imidazole Fluorophores for Selective Hydrazine Detection and Forensic Applications
Source: ACS Omega. 2026 Oct 1;11(40):61442–52. doi: 10.1021/acsomega.6c06606 (PMC13670760; doi:10.1021/acsomega.6c06606)
Supplement: Supplementary file 1 [file ao6c06606_si_001.pdf]

Supplementary Materials

Structure–Driven Intramolecular Charge Transfer Modulation in Imidazole Fluorophores for Selective Hydrazine Detection and Forensic Applications

Yu-Hsin Chen[a], Yu-Hong Chen[a], Wei-Ting Chien[a], Hsin-Chieh Lin[b,c,*], Mei-Yu Yeh[a,*]

[a]Department of Chemistry, Chung Yuan Christian University, No. 200, Zhongbei Rd., Zhongli Dist., Taoyuan City 320314, Taiwan, Republic of China
[b]Department of Materials Science and Engineering, National Yang Ming Chiao Tung University, Hsinchu 300093, Taiwan, Republic of China
[c]Center for Intelligent Drug Systems and Smart Bio-Devices (IDS^{2}B), National Yang Ming Chiao Tung University, Hsinchu 30068, Taiwan, Republic of China
* Corresponding author: myyeh@cycu.edu.tw; hclin45@nycu.edu.tw

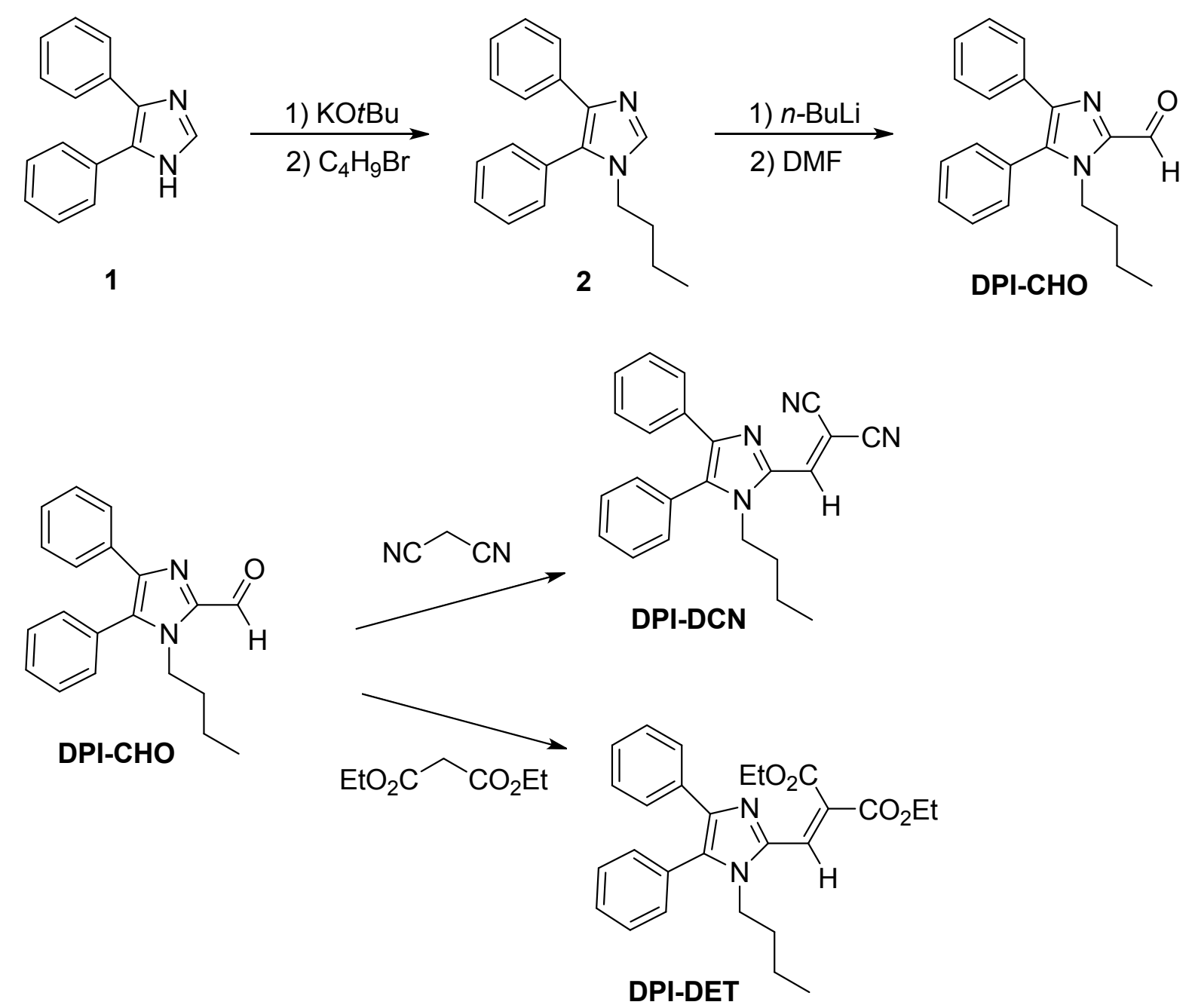

Scheme S1. Synthetic Routes to DPI-DCN and DPI-DET.

Synthesis of compound **2**:

4,5-Diphenylimidazole (4.0 g, 18.2 mmol) and potassium tert-butoxide (2.65 g, 23.6 mmol) were dissolved in anhydrous THF (20 mL) in a 50 mL two-neck round-bottom flask. The reaction mixture was stirred at 0 °C for 1 h, during which a mixture of a pale pink precipitate and a blue solution was observed. 1-Bromobutane (2.56 mL, 23.6 mmol) was then added dropwise at 0 °C, and the reaction mixture was allowed to warm to room temperature and stirred overnight. The reaction was quenched and extracted with dichloromethane (DCM), followed by gravity filtration. The combined organic layers were concentrated under reduced pressure to afford the N-alkylated product (**2**) as a pale yellow solid (4.26 g, 85% yield). ^{1}H NMR (400 MHz, DMSO-d_6): δ= 7.83 (s, 1H), 7.49-7.52 (m, 3H), 7.36-7.38 (m, 4H), 7.10-7.19 (m,3H), 3.76 (t, *J*= 7.3 Hz, 2H), 1.45-2.09 (m, 4H), 0.71 (t, *J*= 7.3 Hz, 3H).

Synthesis of DPI-CHO:

To a solution of compound **2** (0.95 g, 3.44 mmol) in anhydrous THF (8 mL) in a two-neck round-bottom flask under a nitrogen atmosphere at −78 °C was added n-

butyllithium (2.5 M in hexanes, 0.21 mL, 5.17 mmol) dropwise. After stirring for 1 h at −78 °C, DMF (1.47 mL, 1.47 mmol) was added slowly. The reaction mixture was maintained at −78 °C for an additional 1 h, then allowed to warm to room temperature and stirred for 2 h. The reaction was quenched with saturated aqueous NH_4Cl, and the resulting mixture was extracted with ethyl acetate and water. The organic layer was washed with brine (50 mL), dried over anhydrous Na_2SO_4, and concentrated under reduced pressure to afford a yellow oily residue. The crude product was purified by precipitation from a mixture of acetone and diethyl ether to yield DPI-CHO as a yellow solid (0.74 g, 70%). ^{1}H NMR (300 MHz, DMSO-d_6): δ= 7.83 (s, 1H), 7.37-7.51 (m, 8H), 7.10-7.19 (m, 2H), 3.76 (t, *J*= 7.0 Hz, 2H), 1.18-2.00 (m, 4H), 0.71 (t, *J*= 7.0 Hz, 3H).

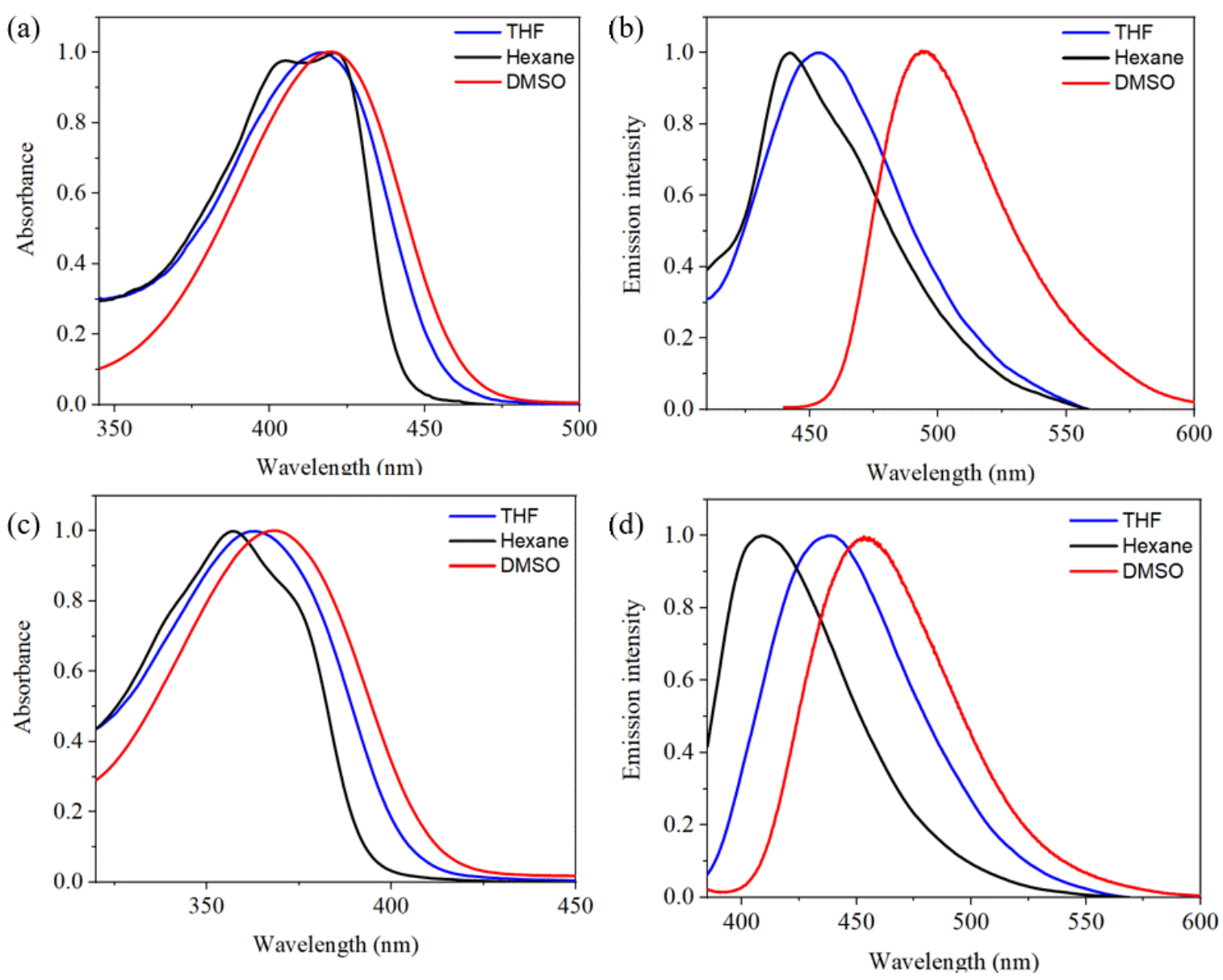

Figure S1. Solvent-dependent photophysical properties of DPI-DCN and DPI-DET: (a) UV–vis absorption spectra of DPI-DCN, (b) fluorescence emission spectra of DPI-DCN, (c) UV–vis absorption spectra of DPI-DET, and (d) fluorescence emission spectra of DPI-DET in hexane, THF, and DMSO.

Table S1. The quantum yields of DPI-DCN, and DPI-DET in solution and solid state.

	**DPI-DCN**	**DPI-DET**
Solution[a,b]	1.02%	1.86%
Solid[b]	13.53%	6.27%

[a]Measured in DMSO; [b]Determined by absolute method.[S1]

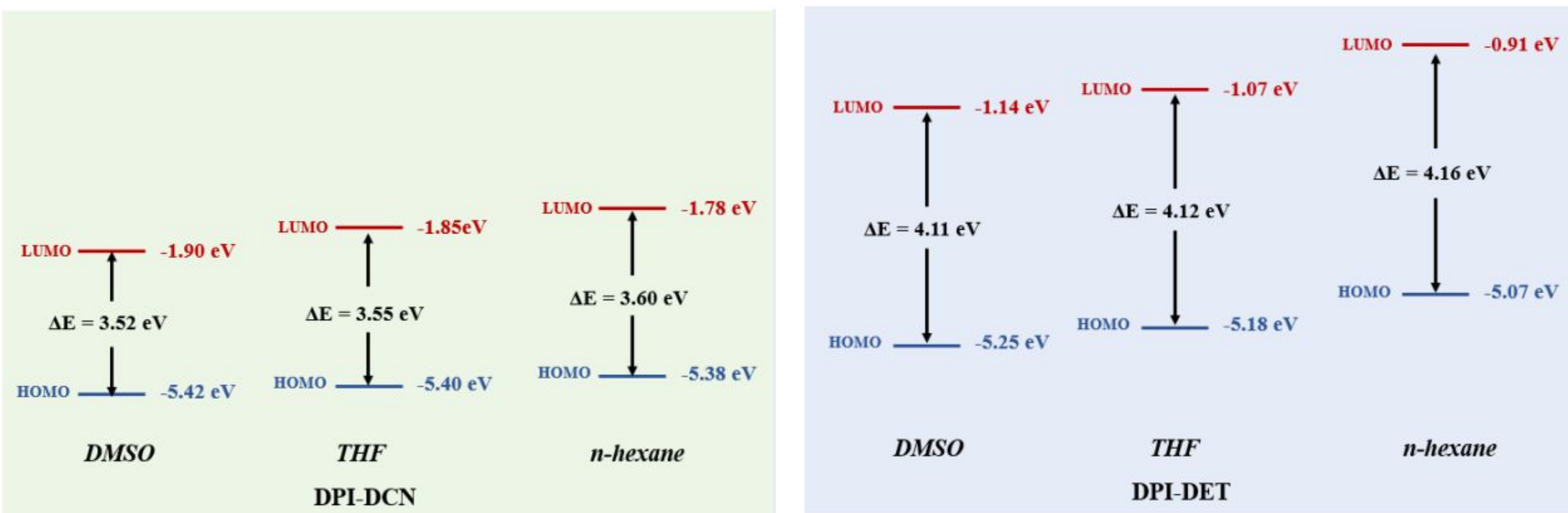

Figure S2. Calculated HOMO–LUMO energy gaps of DPI-DCN and DPI-DET in n-hexane, THF, and DMSO.

Table S2. Experimental and calculated photophysical properties of DPI-DCN and DPI-DET in n-hexane, THF, and DMSO.

	**Experimental**		**Theoretical calculation[a]**	
	**λabs (nm)**	**λem (nm)**	**λabs (nm)**	**λem (nm)**
DPI-DCN/DMSO	420	493	376	467
DPI-DCN/THF	416	451	365	408
DPI-DCN/n-hexane	405	435	349	386
DPI-DET/DMSO	365	453	330	439
DPI-DET/THF	361	437	328	422
DPI-DET/n-hexane	358	409	324	387

[a]CAM-B3LYP/6-31+G(d,p) method with the polarizable continuum model (PCM).

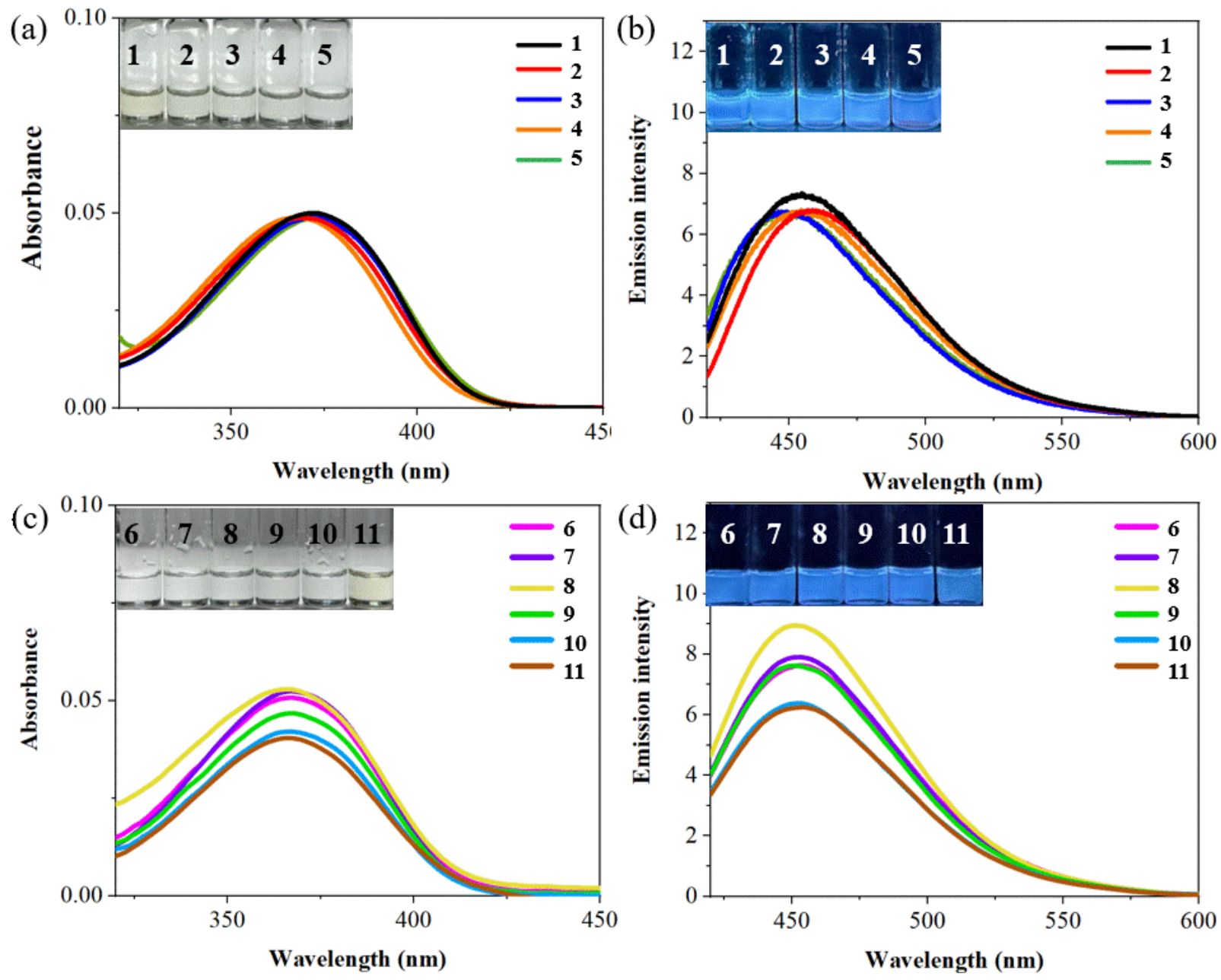

Figure S3 (a, c) Absorption spectra and (b, d) fluorescence spectra of DPI-DET in the presence of 1 equiv of various analytes: 1, blank; 2, N_2H_4; 3, HSO_4^-; 4, ClO_4^-; 5, Cl^-; 6, NH_2OH; 7, NH_3; 8, methylamine; 9, ethylenediamine; 10, phenylhydrazine; and 11, 2,4-dinitrophenylhydrazine. (λ_{ex}= 368 nm; insets show the corresponding optical images of the solutions before and after the addition of each analyte, with the left panels taken under ambient light and the right panels under 365 nm UV irradiation.)

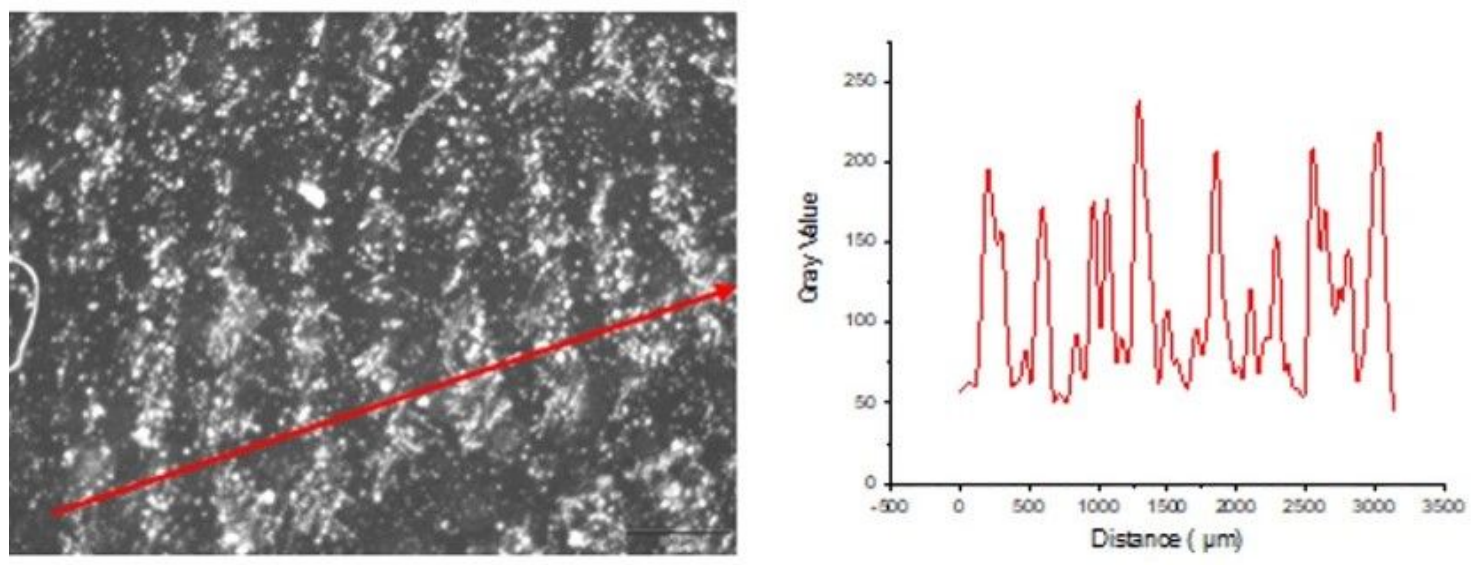

Figure S4. Grayscale values measured on a glass substrate using DPI-DCN powder.

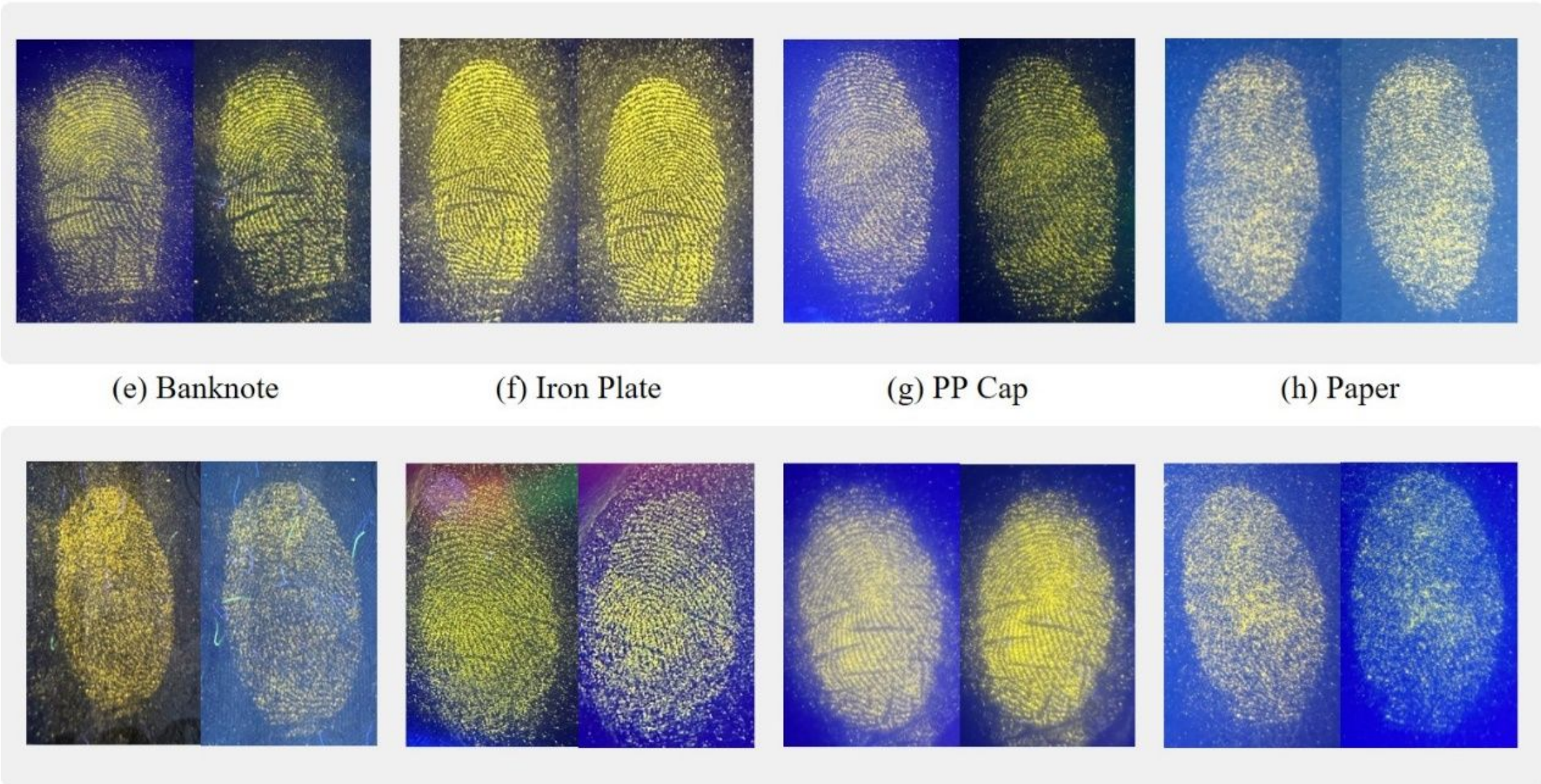

Figure S5. Fluorescence images of LFPs on various substrates indicated by DPI-DCN (left: aged for 1 h; right: aged for four months).

Table S3. Comparative performance of DPI-DCN and previously reported fluorescent materials for latent fingerprint development.

**Component**	**Method**	**Long term**	**LFPs**	**Ref**
CFT	powder dusting	30 days	Level I–III	S2
TPA-10H	solution spraying/ soaking	3 months	Level I–III	S3
Magnetic Nanoparticles	magnetite composite powder	N.A.	Level I–III	S4
PMs+N2H4	solution spraying	30 days	Level I–III	S5
o-CD, *m*-CD, *p*-CD	tripartite mixed powder	N.A.	Level I–III	S6
Redemissive carbon dots	powder dusting	30 days	Level I–III	S7
E-CCDs	powder dusting	N.A.	Level I–II	S8
ADDPh, ADDSi	solution Incubated	N.A.	Level I–II	S9
TCMn	solution	N.A.	Level I–III	S10
IrppyDPBA	solution	N.A.	Level I–III	S11
IMD FT	powder dusting	30 days	Level I–III	S12
DPI-DCN (Our Work)	powder dusting	4 months	Level I–III	/

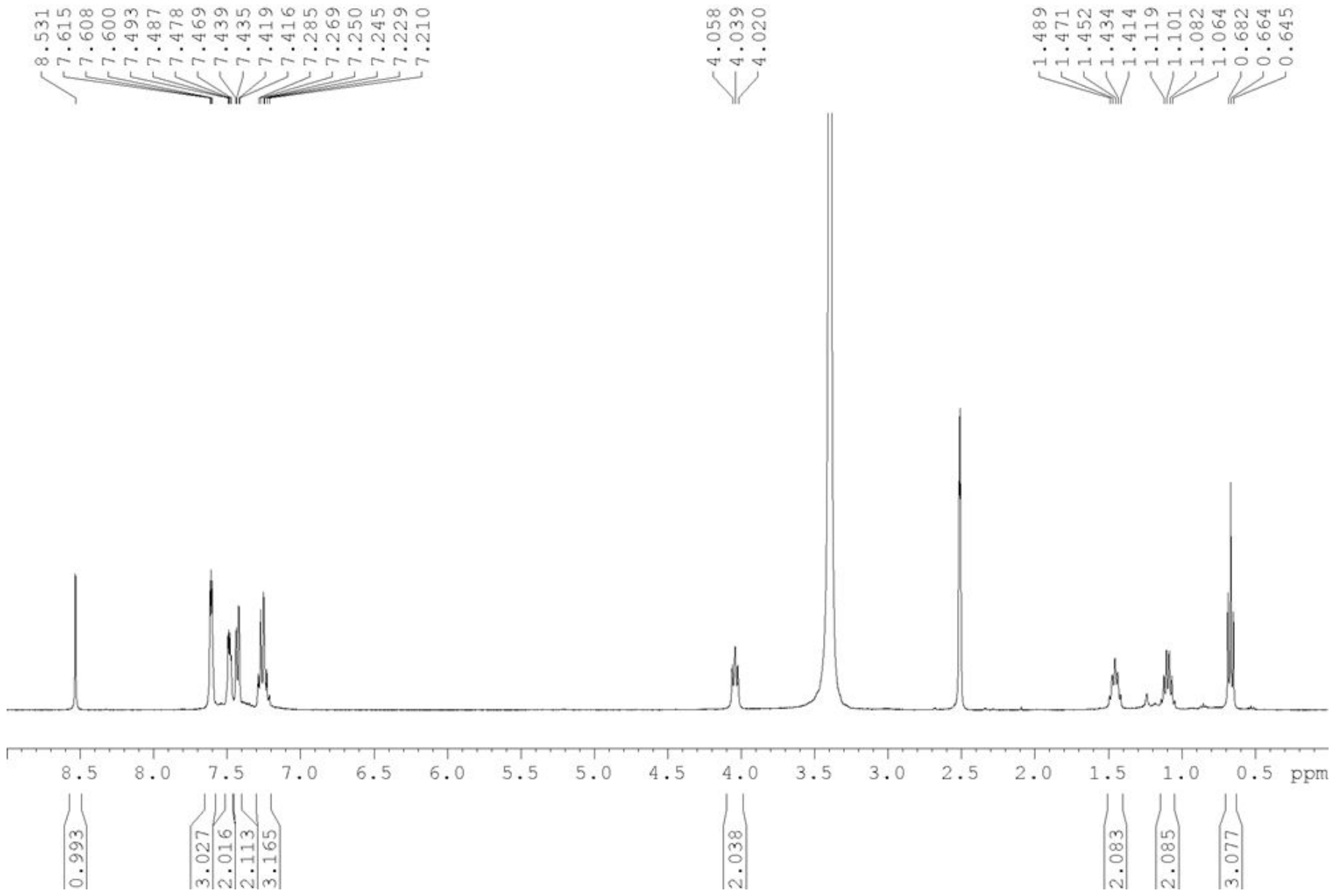

Figure S6. ^{1}H NMR spectrum of DPI-DCN.

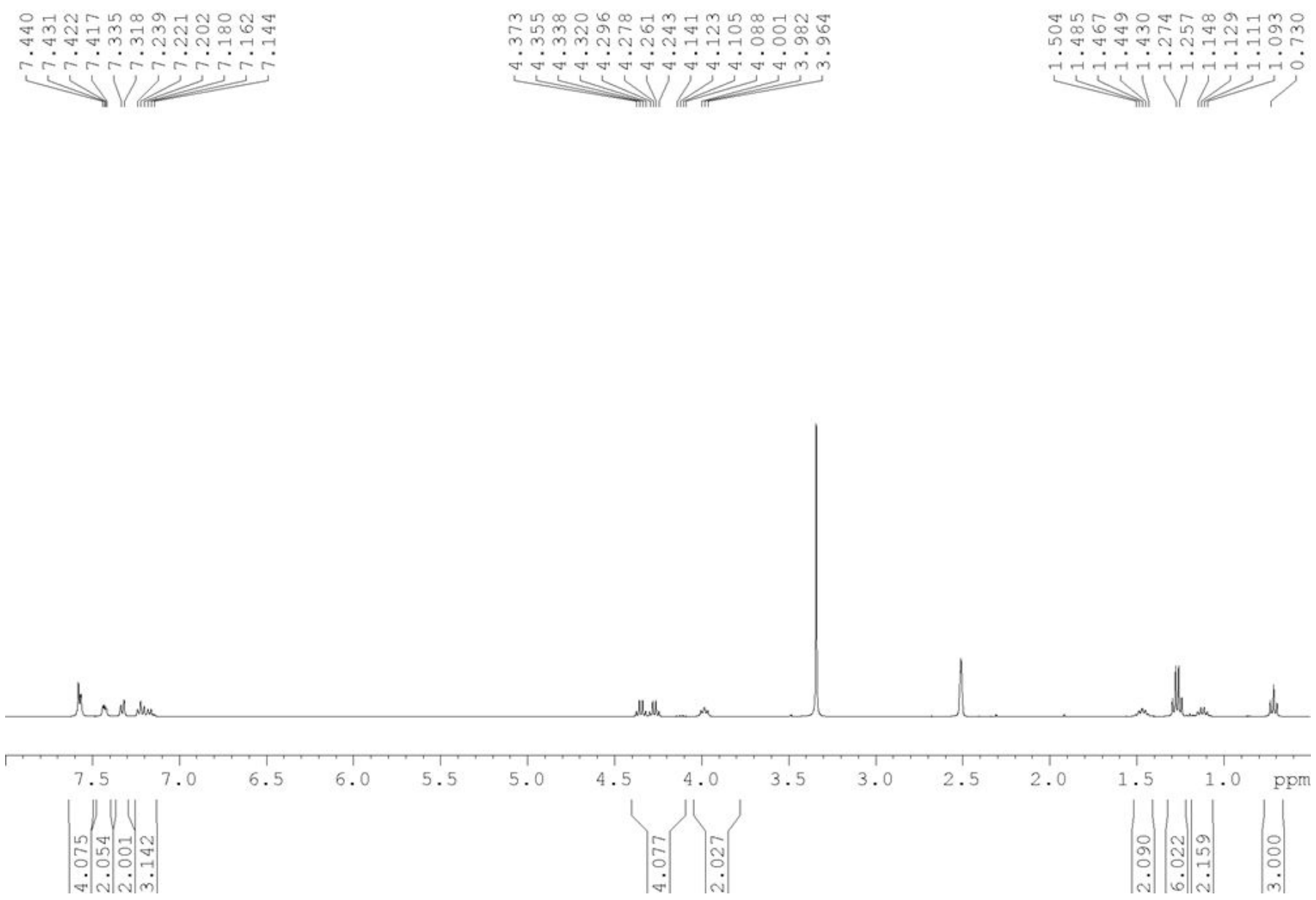

Figure S7. ^{1}H NMR spectrum of DPI-DET.

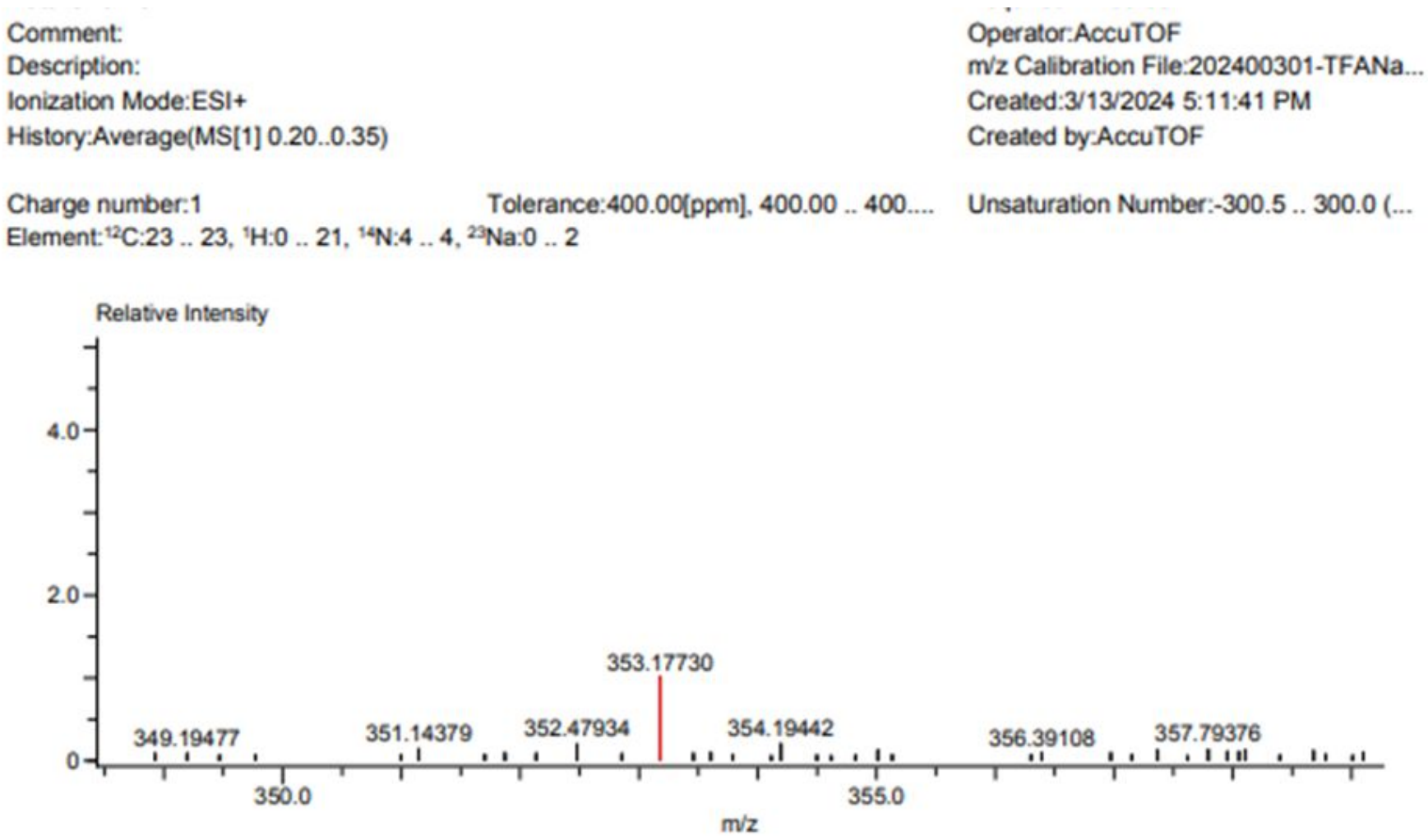

Mass	Intensity	Calc. Mass	Mass Difference [mDa]	Mass Difference [ppm]	Possible Formula
353.17730	508.29	353.17662	0.68	1.93	$^{12}C_{23}{}^{1}H_{21}{}^{14}N_4$

Figure S8. HRMS of DPI-DCN.

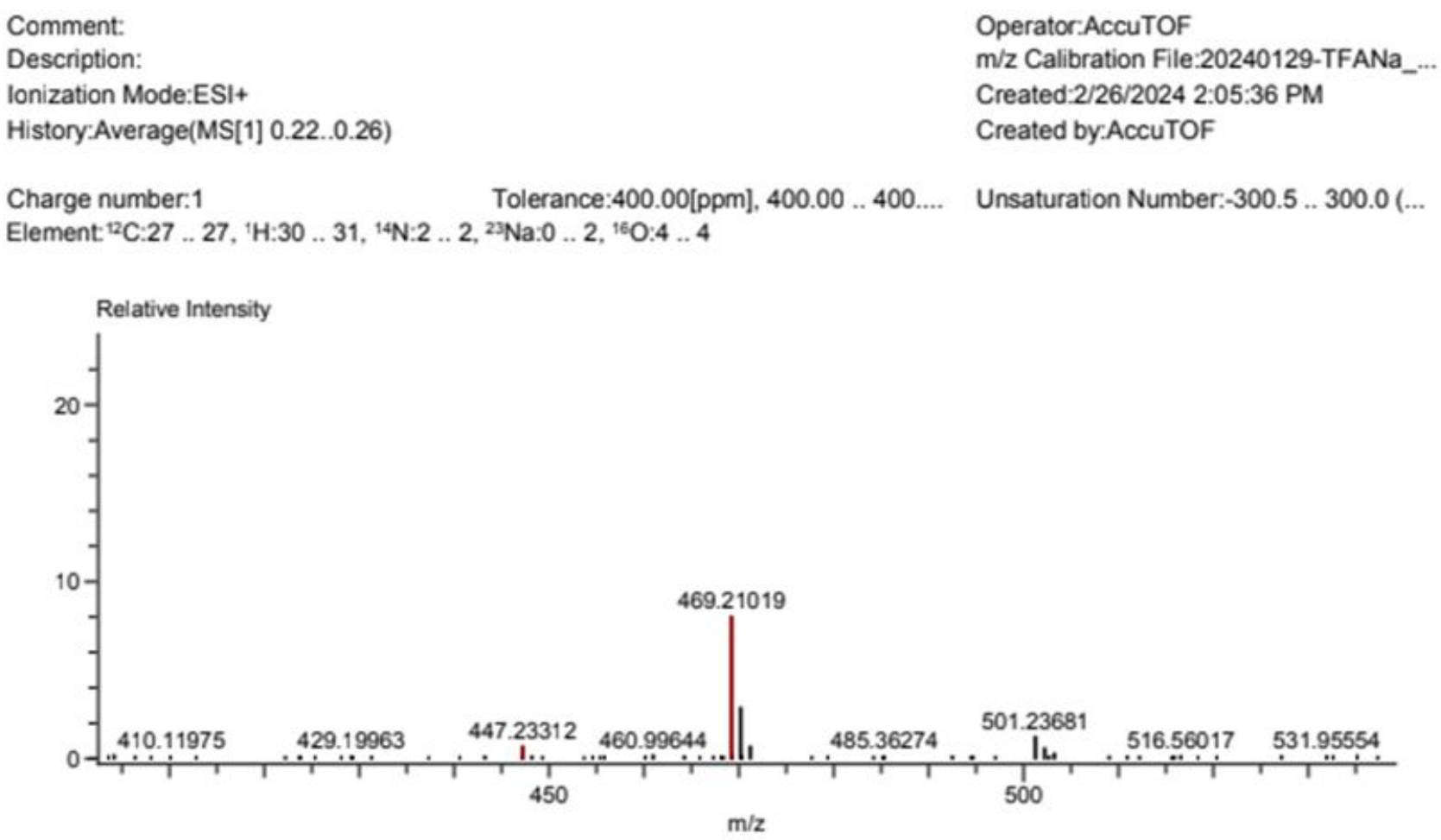

Mass	Intensity	Calc. Mass	Mass Difference [mDa]	Mass Difference [ppm]	Possible Formula
447.23312	841.66	447.22838	4.74	10.60	$^{12}C_{27}{}^{1}H_{31}{}^{14}N_2{}^{16}O_4$
469.21019	9921.50	469.21033	-0.13	-0.28	$^{12}C_{27}{}^{1}H_{30}{}^{14}N_2{}^{23}Na_1{}^{16}O_4$

Figure S9. HRMS of DPI-DET.

Reference

S1 Song, Z.; Zhang, W.; Jiang, M.; Sung, H. H. Y.; Kwok,R. T. K.; Nie, H.; Williams, I. D.; Liu, B.; Tang, B. Z. Synthesis of Imidazole-Based AIEgens with Wide Color Tunability and Exploration of their Biological Applications. *Adv. Funct. Mater.* **2016**, *26*, 824-832.

S2 Duan, L.; Zheng, Q.; Tu, T. Instantaneous High-Resolution Visual Imaging of Latent Fingerprints in Water Using Color-Tunable AIE Pincer Complexes. *Adv. Mater.* **2022**, *34* (35), 2202540.

S3 Wang, Y.-L.; Li, C.; Qu, H.-Q.; Fan, C.; Zhao, P.-J.; Tian, R.; Zhu, M.-Q. Real-Time Fluorescence In Situ Visualization of Latent Fingerprints Exceeding Level 3 Details Based on Aggregation-Induced Emission. *J. Am. Chem. Soc.* **2020**, *142* (16), 7497-7505.

S4 Lee, J.; Lee, C. W.; Kim, J.-M. A Magnetically Responsive Polydiacetylene Precursor for Latent Fingerprint Analysis. *ACS Appl. Mater. Interfaces* **2016**, *8* (9), 6245-6251.

S5 Zou, R.; Yu, Y.; Pan, H.; Zhang, P.; Cheng, F.; Zhang, C.; Chen, S.; Chen, J.; Zeng, R. Cross-Linking Induced Emission of Polymer Micelles for High-Contrast Visualization Level 3 Details of Latent Fingerprints. *ACS Appl. Mater. Interfaces* **2022**, *14* (14), 16746-16754.

S6 Xu, X.; Mo, L.; Li, W.; Li, Y.; Lei, B.; Zhang, X.; Zhuang, J.; Hu, C.; Liu, Y. Red, green and blue aggregation-induced emissive carbon dots. *Chin. Chem. Lett.* **2021**, *32* (12), 3927-3930.

S7 Niu, X.; Song, T.; Xiong, H. Large scale synthesis of red emissive carbon dots powder by solid state reaction for fingerprint identification. *Chin. Chem. Lett.* **2021**, *32* (6), 1953-1956.

S8 Ding, L.; Jin, X.; Gao, Y.; Wu, J.; Ai, T.; Zhou, H.; Chen, X.; Zhang, X.; Chen, W. Facile Preparation Strategy of Novel Carbon Dots with Aggregation-Induced Emission and Room-Temperature Phosphorescence Characteristics. *Adv. Opt. Mater.* **2023**, *11* (5), 2202349.

S9 Suresh, R.; Thiyagarajan, S. K.; Ramamurthy, P. An AIE based fluorescent probe for digital lifting of latent fingerprint marks down to minutiae level. *Sens. Actuators B: Chem.* **2018**, *258*, 184-192.

S10 Li, S.; Wang, L.; Ma, Y.; Zhu, L.; Lin, W. A multifunctional fluorescent molecule with AIE characteristics for SO2 derivatives detection, fluorescence ink and latent fingerprint imaging. *Sens. Actuators B: Chem.* **2022**, *371*, 132595.

S11 Di, L.; Xing, Y.; Yang, Z.; Xia, Z. Ultrabright AIE of Ir(III) complexes achieving expeditious monitoring of oxygen and high-definition development of latent fingerprints. *Sens. Actuators B: Chem.* **2022**, *350*, 130894.

S12 Ravindra, M. K.; Mahadevan, K. M.; Basavaraj, R. B.; Darshan, G. P.; Sharma, S. C.; Raju, M. S.; Vijayakumar, G. R.; Manjappa, K. B.; Yang, D.-Y.; Nagabhushana, H. New design of highly sensitive AIE based fluorescent imidazole derivatives: Probing of sweat pores and anti-counterfeiting applications. *Mater. Sci. Eng. C.* **2019**, *101*, 564-574.
